# Clinical profiles associated with rapidly progressive interstitial lung disease in antisynthetase syndrome: A multicentric cohort study (TYPASS study)

**DOI:** 10.1111/joim.70058

**Published:** 2025-12-10

**Authors:** Maxime Billotte, Thomas Moulinet, Alain Meyer, Houda Camara, Loïs Bolko, Kévin Didier, Sandra Dury, Bernard Bonnotte, Hervé Devilliers, Philippe Bonniaud, Guillaume Beltramo, Julien Campagne, Nadine Magy‐Bertrand, Aurore Chaudier, Simon Valentin, Roland Jaussaud, Paul Decker

**Affiliations:** ^1^ Département de Médecine Interne et Immunologie Clinique CHRU Nancy Vandœuvre‐lès‐Nancy France; ^2^ UMR 7365 CNRS‐Université de Lorraine IMoPA Université de Lorraine Vandœuvre‐lès‐Nancy France; ^3^ Département de Rhumatologie Hôpitaux Universitaires de Strasbourg Strasbourg France; ^4^ Unité de Méthodologie, Datamanagement et Statistiques (UMDS), Département de Recherche Clinique et Innovation CHRU Nancy Vandœuvre‐lès‐Nancy France; ^5^ Département de Rhumatologie CHU Reims Reims France; ^6^ Département de Médecine Interne CHU Reims Reims France; ^7^ Service des Maladies Respiratoires CHU Reims Reims France; ^8^ Département de Médecine Interne et Immunologie Clinique CHU Dijon Dijon France; ^9^ Département de Médecine Interne et Maladies Systémiques CHU Dijon Dijon France; ^10^ Institut Universitaire du Poumon, Centre de Référence Constitutif des Maladies Pulmonaires Rares de l'adultes, Inserm 1231 CTM Université Bourgogne Europe, Centre Hospitalo‐Universitaire Dijon‐Bourgogne Dijon France; ^11^ Service de Médecine Interne UNEOS Metz France; ^12^ Département de Médecine Interne CHU Besançon Besançon France; ^13^ Service de Médecine Interne et Rhumatologie Hôpital Legouest Metz France; ^14^ Département de Pneumologie, CHRU Nancy Vandœuvre‐lès‐Nancy France; ^15^ EA 3450 DevAH—Développement, Adaptation et Handicap Université de Lorraine Vandoeuvre‐lès‐Nancy France

**Keywords:** antisynthetase syndrome, connective tissue disease, interstitial lung disease, myositis, overlap myositis

## Abstract

**Objectives:**

To assess factors associated with rapidly progressive interstitial lung disease (ILD) (RP‐ILD) at time of ILD diagnosis in a multicentric retrospective cohort study of antisynthetase syndrome (ASyS). We used a complementary unsupervised approach, hierarchical clustering, to delineate distinct phenotypes among ASyS patients with ILD.

**Methods:**

A total of 132 patients with ASyS, defined according to the 2024 ACR/European Alliance of Associations for Rheumatology (EULAR) ASyS classification criteria, and ILD, diagnosed by CT scan, were included. RP‐ILD was defined by the presence of respiratory failure at ILD diagnosis or rapid ILD progression during the first 3 months.

**Results:**

In our study, 39% of patients had RP‐ILD at ILD diagnosis. Multivariate logistic regression analysis with cluster‐robust SE identified the factors associated with RP‐ILD at ILD diagnosis as male sex (aOR = 9.7 [1.6–59.5], *p* = 0.006), fever (aOR = 128 [12.6–1300], *p* < 0.001), organizing pneumonia (OP) pattern (aOR = 66.8 [3.4–1316], *p* = 0.006), and pleural effusion (aOR = 20.2 [1.1–373], *p* = 0.04), whereas RP‐ILD was associated with lower likelihood of severe muscle disease (aOR = 0.004 [0.0001–0.13], *p* = 0.002). Clustering analysis identified four distinct groups: Cluster 1 (*n* = 62) included patients with systemic presentation, non‐RP‐ILD at ILD diagnosis, and anti‐Jo‐1 antibodies with good prognosis; Cluster 2 (*n* = 40) included older age patients with more RP‐ILD at ILD diagnosis, pleuropericarditis, and a higher mortality rate.

**Conclusion:**

Fever, pleural effusion, and OP pattern were independently associated with RP‐ILD in ASyS patients. Unsupervised cluster analysis identified a severe inflammatory phenotype in ASyS patients with ILD.

## Graphical abstract

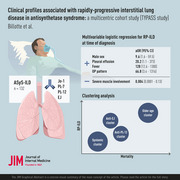

**Abbreviations**
ACR: American College of Rheumatology
ARS: aminoacyl‐tRNA‐synthetase
ASyS: antisynthetase syndrome
CTD: connective tissue disease
DLC_O_
: diffusion capacity of the lung for carbon monoxide
EMG: electromyography
EULAR: European Alliance of Associations for Rheumatology
FVC: forced vital capacity
IIM: idiopathic inflammatory myopathy
ILD: interstitial lung disease
MCA: multiple correspondence analysis
MRC: Medical Research Council
NSIP: nonspecific interstitial pneumonia
OP: organizing pneumonia
mPAP: mean pulmonary arterial pressure
PFT: pulmonary function test
PH: pulmonary hypertension
RP‐ILD: rapidly progressive interstitial lung disease
TLC: total lung capacity
UIP: usual interstitial pneumonia

## Introduction

Antisynthetase syndrome (ASyS) is an idiopathic inflammatory myopathy (IIM), a group of systemic autoimmune disorders affecting primarily muscle tissue [1, 2, 3]. It is characterized by anti‐aminoacyl‐tRNA‐synthetase (anti‐ARS) antibodies. Since the identification of anti‐histidyl‐tRNA‐synthetase (anti‐Jo‐1) antibodies in 1980, several other anti‐ARS antibodies have been described [4, 5, 6, 7]. These antibodies are mutually exclusive and serve as diagnostic criteria for ASyS [8, 9].

ASyS presents a heterogeneous disease spectrum [10]. Anti‐ARS antibodies are associated with clinical phenotypes: Anti‐Jo‐1‐positive patients often exhibit a systemic phenotype with myositis, polyarthritis, and interstitial lung disease (ILD), whereas anti‐PL‐7 or anti‐PL‐12‐positive patients more frequently present with predominant or isolated ILD, particularly at disease onset [11, 12]. ASyS‐associated ILD (ASyS‐ILD) is the leading cause of mortality in these patients [12, 13, 14]. In large French and American cohorts, patients with anti‐PL‐7 and anti‐PL‐12 antibodies had worse forced vital capacity (FVC) and diffusion capacity of the lung for carbon monoxide (DL_CO_) at diagnosis and poorer survival compared to anti‐Jo‐1‐positive patients [11, 15]. However, several other cohorts did not observe associations between anti‐ARS antibodies and pulmonary function test (PFT) results at diagnosis [16, 17, 18, 19, 20]. Rapidly progressive ILD (RP‐ILD), a severe subset of ILD observed in IIMs, is characterized by rapid deterioration of respiratory symptoms over days to weeks, often leading to respiratory failure and high mortality [21]. A recent Chinese study on RP‐ILD did not find an association with anti‐ARS antibodies [22]. Although anti‐ARS antibodies offer valuable diagnostic insights, they do not fully explain the diverse clinical phenotypes in ASyS, especially concerning ILD outcomes at diagnosis. Additionally, most studies evaluating ILD outcomes in ASyS included all patients, regardless of ILD presence, potentially confounding ILD‐specific outcome assessments. Therefore, further research is necessary to elucidate the factors associated with ILD outcomes at diagnosis.

This study aimed to identify factors associated with RP‐ILD at ILD diagnosis. Given the disease's heterogeneity, we employed a complementary unsupervised approach, specifically hierarchical clustering, to delineate distinct phenotypes among ASyS patients with ILD, without predefined assumptions.

## Patients and methods

### Study population

We conducted a multicenter cohort study in seven reference and competence centers for managing systemic autoimmune and inflammatory disorders in northeast France.

Inclusion criteria were as follows: (1) adults, (2) patients with definite ASyS according to the 2024 ACR/European Alliance of Associations for Rheumatology (EULAR) proposed classification criteria [8], (3) positivity for anti‐Jo‐1, anti‐PL‐7, anti‐PL‐12, anti‐EJ, or anti‐OJ antibodies, and (4) presence of ILD on high‐resolution CT (HRCT)—based on thoracic radiologist reports—at any time during the disease course irrespective of dyspnea/PFT abnormalities. To avoid including non‐ASyS patients, patients with multiple or rare anti‐ARS antibodies were not included due to the uncertainty regarding their antibody profile and the limited diagnostic performance of laboratory tests for their detection, respectively. Additionally, patients with substantial missing data at ILD diagnosis, which hinder the assessment of ILD outcomes, were excluded.

Anti‐ARS antibodies were detected at diagnosis via immunoprecipitation or ELISA for anti‐Jo‐1 and line/dot immunoblotting for others (EUROLINE Autoimmune Inflammatory Myopathies 16 Ag, Lubeck, Germany; D‐tek BlueDiver Dot Polymyositis/Scleroderma12 IgG, Mons, Belgium).

Associated connective tissue diseases (CTDs), such as rheumatoid arthritis, systemic lupus, Sjögren's syndrome, and systemic sclerosis, were defined using international classification criteria [23, 24, 25, 26].

### ILD outcomes

Due to the lack of consensus on RP‐ILD definition and to comprehensively identify patients with this phenotype, RP‐ILD was defined as respiratory failure at ILD diagnosis (arterial partial pressure of oxygen (PaO2) <60 mmHg or oxygen therapy) without any other causes (e.g., cardiac insufficiency, embolism, or infection) or rapid ILD progression within 3 months after diagnosis—adapted from Wu et al.*—*with at least two of (1) interstitial lesions extension on HRCT, (2) worsening dyspnea, and (3) FVC relative decline ≥5% predicted [22].

ASyS‐ILD relapse was defined by new bilateral ground‐glass opacities on HRCT after initial remission, requiring immunosuppression intensification, with no evidence of infection or other causes like cardiac insufficiency [27]. All patients with suspected ILD relapses (new or worsening clinical symptoms, PFTs deterioration) underwent HRCT.

Pulmonary hypertension (PH) was suspected with peak tricuspid regurgitation velocity >2.8 m/s and/or indirect signs of chronic pulmonary heart disease on echocardiography and confirmed by mean pulmonary arterial pressure ≥25 mmHg on right heart catheterization according to current practice guidelines at the time of the study design [28].

Chronic respiratory failure was defined as PaO2 <70 mmHg at rest on ambient air and confirmed at least 2 weeks apart.

### Definitions of extra‐respiratory variables

Muscle involvement was defined as creatine phosphokinase (CPK) ≥2× normal and/or MRI inflammatory lesions and/or electromyography (EMG)‐detected myopathy and/or myositis on biopsy. Severe muscle involvement was defined as CPK ≥10× normal, Medical Research Council (MRC) score ≤3/5 in proximal muscles, and/or severe dysphagia as determined by the physician.

Microvascular involvement included Raynaud's phenomenon, distal ischemic lesions (ulcers or necrosis), or nailfold capillaroscopy‐detected organic microangiopathy. Organic microangiopathy was defined by at least one of the following findings: nonspecific capillary morphological variations, dilated/giant capillaries, microhemorrhages, abnormal capillary shapes, or reduced capillary density [29].

Typical dermatomyositis lesions included Gottron's papules, periungual erythema, heliotrope rash, and the shawl/holster signs.

Cancer‐associated myositis was defined as cancer within 3 years before or after ASyS diagnosis.

### Data collection

Clinical, laboratory, imaging, and PFT data were retrospectively collected at ILD diagnosis from patient medical records. Longitudinal ILD outcomes (relapse, PH, chronic respiratory failure, overall, or transplant‐free survival) and treatments were collected from ILD diagnosis to the last follow‐up at the time of inclusion in the study. PFTs, clinical findings, and HRCTs were systematically reviewed to identify ILD relapses. HRCT ILD patterns were based on reports by trained chest radiologists.

### Ethical policy

In accordance with French legislation, written informed consent was unnecessary, but patients received an information note with a non‐opposition form. The study protocol was approved by the local ethics committee of CHRU Nancy (approval number 315) and registered on ClinicalTrials.gov (NCT04924465).

### Statistical analysis

Quantitative data are shown as medians and interquartile range and compared using Student's *t*‐test or the Mann–Whitney *U*‐test. Categorical variables are expressed as numbers and percentages and compared with *χ* ^2^ or Fisher's exact test.

The extent of missing data was quantified by variable. Quantitative missing data were imputed using MICE algorithm.

Candidate baseline variables for predicting RP‐ILD at diagnosis were selected based on expert opinion, including sex, age, body mass index, smoking status, overlapping CTD, cancer‐associated myositis, clinical manifestations at ASyS diagnosis (skin, microvascular, joint, muscle, and cardiac involvement; fever; pleural effusion), ILD pattern (usual interstitial pneumonia [UIP], nonspecific interstitial pneumonia, organizing pneumonia [OP]), and antibody status (anti‐Jo‐1, anti‐PL‐7, anti‐PL‐12, anti‐EJ, anti‐Ro52). In multivariable logistic regression models with cluster‐robust standard errors (to account for within‐center clustering), variables with *p* ≤ 0.10 in univariable analyses were included. Dyspnea scores, PFTs, and blood gas results were excluded, as they form part of the RP‐ILD definition and are closely related to pulmonary severity (risk of multicollinearity). Except for two patients who presented with acute respiratory failure at the time of ILD diagnosis, all others were followed for at least 3 months post‐ILD diagnosis, with no censored observations regarding RP‐ILD. The final model exhibited acceptable overall fit, with residuals largely well‐behaved and no major signs of global misfit. Exploratory extensions incorporating polynomial terms, splines, and interactions did not significantly improve model fit.

For survival analyses, Cox proportional hazards models with cluster‐robust standard errors were used. The following variables with *p* ≤ 0.10 in univariable analyses were considered for multivariable modeling, after excluding those lacking clinical relevance: sex, age, cancer‐associated myositis, clinical manifestations at ASyS diagnosis (muscle and cardiac involvement; pleural effusion), UIP pattern, anti‐PL‐7 antibodies, RP‐ILD, and number of treatments used. The final models demonstrated reasonable global fit, and Schoenfeld residuals indicated no major violations of the proportional hazards assumption. Alternative model specifications (e.g., inclusion of nonlinear terms or interaction effects) did not materially enhance fit.

To explore interrelationships among variables at diagnosis and during follow‐up, we employed an unsupervised clustering approach combining multiple correspondence analysis (MCA) and hierarchical clustering. Variables were selected based on reports from published literature, for their clinical relevance and ability to discriminate patient phenotypes (Table S1) [22, 30]. First, MCA reduced dimensionality and allowed graphical assessment of patient distributions according to shared characteristics. The factor map highlighted the main association patterns, with Dimensions 1 and 2 cumulatively explaining 20.9% of the total variance (Fig. S1). Second, hierarchical clustering applied to the MCA coordinates identified patient groups with similar profiles. The optimal number of clusters was determined by evaluating within‐cluster sum of squares and average silhouette width to balance compactness and separation. The resulting dendrogram visually represented hierarchical patient relationships, with the *y*‐axis indicating fusion height and the *x*‐axis showing ASyS‐ILD patients included in the analysis.

Statistical testing was performed at a two‐tailed α level of 0.05. Analyses were performed using R (v4.3.0).

## Results

### General characteristics

**Fig. 1:**
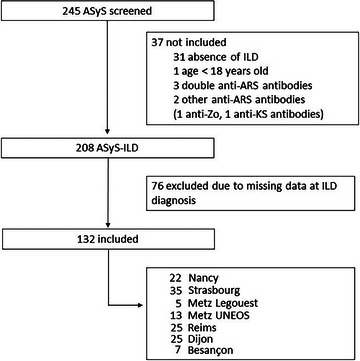
Flowchart of patient inclusion. Alt text: Flowchart illustrating the study design: number of antisynthetase syndrome (ASyS) patients screened, diagnosed with interstitial lung disease (ILD), included in the study, excluded from participation, and reasons for exclusion. Anti‐ARS antibodies, anti‐aminoacyl‐tRNA‐synthetase antibodies; ASyS, antisynthetase syndrome; ASyS‐ILD, antisynthetase syndrome‐associated interstitial lung disease; ILD, interstitial lung disease.

Among 245 ASyS patients, 132 patients with ASyS‐ILD who were diagnosed between January 1994 and December 2023 were included in this study (Fig. 1). The median age was 55 (47–65) years, and 69% of the patients were female. An associated CTD was reported in 18% of patients, mainly Sjogren's syndrome, and 14% had cancer‐associated myositis (Table 1). With the exception of sex, age, and hydroxychloroquine use, no other differences were observed between patients with and without associated CTD (Table S2).

**Table 1:** Characteristics of patients with and without rapidly progressive interstitial lung disease (RP‐ILD) at time of interstitial lung disease (ILD) diagnosis.

Variable	Total population (*N* = 132)	Non‐RP‐ILD (*N* = 81)	RP‐ILD (*N* = 51)	*p* value
**Demographics**				
Female, *n* (%)	91/132 (69)	**62/81 (77)**	**29/51 (57)**	**0.017**
Age, years^a^	55 (47–65)	54 (46–65)	58 (52–67)	0.06
BMI, kg/m^2^ ^a^	27 (25–31)	**26 (23**–**29)**	**28 (25**–**32)**	**0.019**
Former or current smokers, *n* (%)	68/120 (57)^b^	40/75 (53)^c^	28/45 (62)^d^	0.3
Comorbidities, *n* (%)				
Cancer‐associated myositis	18/132 (14)	8/81 (10)	10/51 (20)	0.11
Overlapping connective tissue disease	24/132 (18)	18/81 (22)	6/51 (12)	0.13
Systemic lupus	4/132 (3)	4/81 (5)	0/51 (0)	0.2
Gougerot‐Sjögren syndrome	16/132 (12)	12/81 (15)	4/51 (8)	0.2
Systemic sclerosis	1/132 (1)	1/81 (1)	0/51 (0)	>0.9
Rheumatoid arthritis	7/132 (5)	5/81 (6)	2/51 (4)	0.7
**Clinical manifestations at ASyS diagnosis**				
Skin involvement, *n* (%)	51/132 (39)	35/81 (43)	16/51 (31)	0.2
Mechanic's hands	41/132 (31)	28/81 (35)	13/51 (25)	0.3
Typical dermatomyositis signs	21/132 (16)	13/81 (16)	8/51 (16)	>0.9
Microvascular involvement, *n* (%)	47/132 (36)	33/81 (41)	14/51 (27)	0.12
Raynaud's phenomenon	34/132 (26)	**26/81 (32)**	**8/51 (16)**	**0.036**
Nailfold capillaroscopy abnormalities	18/48 (39)^b^	13/30 (43)^c^	5/16 (31)^d^	0.4
Joint involvement, *n* (%)	78/132 (59)	50/81 (62)	28/51 (55)	0.4
Arthralgia	77/132 (58)	49/81 (60)	28/51 (55)	0.5
Synovitis	40/131 (31)^b^	28/81 (35)	12/50 (24)^d^	0.2
Erosions (radiography)	3/33 (9.1)^b^	3/25 (12)^c^	0/8 (0)^d^	0.6
Muscle involvement, *n* (%)	67/132 (51)	45/81 (56)	22/51 (43)	0.2
Muscle weakness (MRC ≤ 3)	24/130 (18)^b^	18/80 (23)^c^	6/50 (12)^d^	0.13
Dysphagia	5/132 (4)	3/81 (4)	2/51 (4)	>0.9
Severe muscle involvement	36/132 (27)	**27/81 (33)**	**9/51 (18)**	**0.049**
Fever, *n* (%)	44/131 (34)^b^	**13/81 (16)**	**31/50 (62)** ^d^	**<0.001**
Pericarditis, *n* (%)	12/131 (9)^b^	6/81 (7)	6/50 (12)^d^	0.5
Myocarditis, *n* (%)	2/131 (2)^b^	1/81 (1)	1/50 (2)^d^	>0.9
**Antibodies at ASyS diagnosis**				
Anti‐Jo‐1, *n* (%)	80/132 (61)	53/81 (65)	27/51 (53)	0.2
Anti‐PL‐7, *n* (%)	21/132 (16)	11/81 (14)	10/51 (20)	0.4
Anti‐PL‐12, *n* (%)	21/132 (16)	12/81 (15)	9/51 (18)	0.7
Anti‐EJ, *n* (%)	10/132 (8)	5/81 (6)	5/51 (10)	0.5
Anti‐Ro52 (TRIM21), *n* (%)	40/75 (53)^b^	26/45 (58)^c^	14/30 (47)^d^	0.3
**Laboratory indicators at ILD diagnosis**				
C‐reactive protein, mg/L^a^	24 (10–53)	**18 (4**–**33)**	**45 (23**–**111)**	**<0.001**
Ferritin, µg/L^a^	323 (99–551)	304 (85–553)	342 (230–521)	0.7
Creatine phosphokinase, IU/L^a^	529 (111–1781)	**1009 (188**–**3075)**	**277 (61**–**897)**	**<0.001**
**ILD outcomes at ILD diagnosis**				
Dyspnea on mMRC scale, *n* (%)				**<0.001**
0	19/104 (18)^b^	18/62 (29)^c^	1/42 (2)^d^	
1	15/104 (14)^b^	15/62 (24)^c^	0/42 (0)^d^	
2	23/104 (22)^b^	18/62 (29)^c^	5/42 (12)^d^	
3	20/104 (19)^b^	9/62 (15)^c^	11/42 (26)^d^	
4	27/104 (26)^b^	2/62 (3)^c^	25/42 (60)^d^	
Cough, *n* (%)	75/127 (59)^b^	43/78 (55)^c^	32/49 (65)^d^	0.3
ILD pattern, *n* (%)				
UIP	8/71 (11)^b^	6/45 (13)^c^	2/26 (8)^d^	0.7
NSIP	57/71 (80)^b^	38/45 (84)^c^	19/26 (73)^d^	0.2
OP	11/71 (15)^b^	4/45 (9)^c^	7/26 (27)^d^	0.09
Pleural effusion, *n* (%)	22/123 (18)^b^	**4/75 (5)** ^c^	**18/48 (38)** ^d^	**<0.001**
Pulmonary function test results				
PaO_2_, mmHg^a^	71 (6–90)	**76 (69**–**93)**	**67 (58**–**76)**	**0.005**
FVC, % predicted^a^	72 (56–85)	**74 (65**–**88)**	**64 (48**–**74)**	**<0.001**
TLC, % predicted^a^	76 (59–89)	**81 (66**–**93)**	**61 (53**–**72)**	**<0.001**
DL_CO_, % predicted^a^	50 (42–63)	**58 (46**–**71)**	**44 (32**–**47)**	**<0.001**
DL_CO_/VA, % predicted^a^	73 (64–86)	73 (64–92)	72 (60–81)	0.3
Rapidly progressive ILD, *n* (%)	51/132 (39)	–	–	–
Respiratory failure, *n* (%)	46/132 (35)	0/81 (0)	46/51 (90)	<0.001
Worsening ILD in the first 3 months, *n* (%)	5/132 (4)	0/81 (0)	5/51 (10)	0.02
**ILD outcomes during follow‐up**				
Follow‐up time, months^a^	49 (24–86)	55 (27–93)	44 (18–73)	0.16
Number of ILD relapses^a^	1 (0–2)	0 (0–2)	1 (0–2)	0.2
Confirmed pulmonary hypertension, *n* (%)	11/132 (8)	8/81 (10)	3/51 (6)	0.5
Chronic respiratory failure, *n* (%)	29/131 (22)^b^	**11/81 (14)**	**18/50 (36)** ^d^	**0.003**
Long‐term oxygen therapy, *n* (%)	27/130 (21)^b^	**11/80 (14)** ^c^	**16/50 (32)** ^d^	**0.013**
Lung transplantation, *n* (%)	1/132 (1)	1/81 (1)	0/51 (0)	>0.9
Death, *n* (%)	27/132 (20)	13/81 (16)	14/51 (27)	0.11
**Treatments used during follow‐up**				
Number of treatments used during follow‐up^a^	3 (2–4)	3 (2–4)	3 (2–4)	0.3
Corticosteroids, *n* (%)	125/132 (95)	75/81 (93)	50/51 (98)	0.2
Hydroxychloroquine, *n* (%)	13/132 (10)	**12/81 (15)**	**1/51 (2)**	**0.016**
Methotrexate, *n* (%)	54/132 (41)	37/81 (46)	17/51 (33)	0.2
Azathioprine, *n* (%)	35/132 (27)	20/81 (25)	15/51 (29)	0.5
Cyclophosphamide, *n* (%)	36/132 (27)	**14/81 (17)**	**22/51 (43)**	**0.001**
Mycophenolate mofetil, *n* (%)	54/132 (41)	35/81 (43)	19/51 (37)	0.5
Rituximab, *n* (%)	50/132 (38)	**25/81 (31)**	**25/51 (49)**	**0.036**
CNI, *n* (%)	12/132 (9)	8/81 (10)	4/51 (8)	0.8
IVIg, *n* (%)	24/132 (18)	16/81 (20)	8/51 (16)	0.6
Antifibrotic drugs, *n* (%)	5/132 (4)	4/81 (5)	1/51 (2)	0.6

*Note*: Bold font indicates statistically significant results (*p* < 0.05).

Abbreviations: ASyS, antisynthetase syndrome; BMI, body mass index; CNI, calcineurin inhibitor; DL_CO_, diffusion capacity of the lung for carbon monoxide; DL_CO_/VA, diffusion capacity of the lung for carbon monoxide per lung volume; FVC, forced vital capacity; ILD, interstitial lung disease; IVIg, intravenous immunoglobulin; mMRC, modified Medical Research Council dyspnea scale; MRC, Medical Research Council muscle testing scale; NSIP, nonspecific interstitial pneumonia; OP, organized pneumonia; PaO2, arterial partial pressure of oxygen; TLC, total lung capacity; UIP, usual interstitial pneumonia.

^a^ Data are expressed as medians with interquartile ranges (IQRs).

^b^ *N* ≠ 132 due to missing data.

^c^ *N* ≠ 81 due to missing data.

^d^ *N* ≠ 51 due to missing data.

Regarding the anti‐ARS antibodies at ASyS diagnosis, 61% of patients had anti‐Jo‐1 antibodies, 16% had anti‐PL‐7 antibodies, 16% had anti‐PL‐12 antibodies, 8% had anti‐EJ antibodies, and no patients had anti‐OJ antibodies. More than half of the patients were tested for anti‐Ro52 antibodies, and 53% of the patients were positive for these antibodies.

The main extrapulmonary manifestations at ASyS diagnosis were joint manifestations (59%), muscle involvement (51% of patients, with severe muscle involvement in 27%), skin manifestations (39%), and microvascular involvement (36%). Fever was reported in 34% of patients. An isolated ILD was observed in 14 patients (11%) at the time of initial diagnosis.

### Rapidly progressive ILD at time of ILD diagnosis

ILD at the time of ASyS diagnosis was reported in 94% of patients. For the 11 patients diagnosed with ILD during ASyS follow‐up (9 with anti‐Jo‐1 antibodies, 2 with anti‐PL‐12 antibodies), the median time between ASyS diagnosis and ILD diagnosis was 54 (34−297) days, and 7 patients were diagnosed in the first 3 months after ASyS diagnosis. Among ASyS‐ILD patients, 39% had RP‐ILD at ILD diagnosis, mainly due to respiratory failure with hypoxemia; 42 patients (32%) received oxygen therapy, and 8 patients (6%) required invasive mechanical ventilation.

When comparing the distribution of anti‐ARS antibodies between RP‐ILD patients and non‐RP‐ILD patients at ILD diagnosis, anti‐Jo‐1 antibodies tended to be less common in RP‐ILD patients (Table 1). Given the importance of antibodies in defining clinical phenotypes and the ongoing debate about their prognostic value, we performed an additional analysis comparing ILD outcomes in patients with different anti‐ARS antibodies, under the hypothesis of a potential difference between anti‐Jo1 and non‐anti‐Jo1 patients (Table S3). Patients with anti‐Jo‐1 antibodies had a higher FVC and less dyspnea at ILD diagnosis compared to non‐anti‐Jo‐1 patients.

The factors associated with RP‐ILD at ILD diagnosis in the univariate logistic regression analysis were sex, BMI, fever, CRP level, pleural effusion, and OP pattern (Table 2). RP‐ILD at ILD diagnosis was associated with less frequent Raynaud's phenomenon, less severe muscle involvement, and lower CPK levels. In the multivariate analysis, the factors associated with RP‐ILD at ILD diagnosis included fever (aOR = 128.1 [12.6−1300]; *p* < 0.001), male sex (aOR = 9.63 [1.56−59.5]; *p* = 0.006), OP pattern (aOR = 66.8 [3.39−1316]; *p* = 0.05), and pleural effusion (aOR = 20.2 [1.09−373]; *p* = 0.04), whereas RP‐ILD was associated with lower likelihood of severe muscle involvement (aOR = 0.004 [0.0001−0.13]; *p* = 0.002) (Table 2).

**Table 2:** Factors associated with rapidly progressive interstitial lung disease (RP‐ILD) at time of interstitial lung disease (ILD) diagnosis.

Variable	Univariate analysis		Multivariate analysis	
	OR (95% CI)	*p* value	aOR (95% CI)	*p* value
**Demographics**				
Male sex	**2.48 (1.17–5.32)**	**0.018**	**9.63 (1.56–59.5)**	**0.006**
Age	1.02 (1.00–1.05)	0.053	1.01 (0.95–1.08)	0.7
BMI	**1.09 (1.01–1.18)**	**0.02**	0.97 (0.78–1.21)	0.79
Former or current smokers	1.44 (0.68–3.1)	0.3	–	–
Cancer‐associated myositis	2.23 (0.82–6.26)	0.12	–	–
Overlapping connective tissue disease	0.47 (0.16–1.21)	0.12	–	–
**Clinical manifestations at ASyS diagnosis**				
Skin involvement	0.6 (0.28–1.24)	0.2	–	–
Mechanic's hands	0.65 (0.29–1.39)	0.3	–	–
Microvascular involvement	0.55 (0.25–1.16)	0.12	–	–
Raynaud's phenomenon	**0.39 (0.15–0.92)**	**0.031**	0.32 (0.04–2.99)	0.32
Joint involvement	0.75 (0.37–1.54)	0.4	–	–
Muscle involvement	0.61 (0.3–1.23)	0.2	–	–
Severe muscle involvement	**0.43 (0.17–0.98)**	**0.044**	**0.004 (0.0001–0.13)**	**0.002**
Fever	**8.53 (3.83–20.1)**	**<0.001**	**128.1 (12.6–1300)**	**<0.001**
Cardiac involvement (pericarditis and/or myocarditis)	1.7 (0.5–5.76)	0.4	–	–
**Antibodies at ASyS diagnosis**				
Anti‐Jo‐1	0.59 (0.29–1.22)	0.2	–	–
Anti‐PL‐7	1.55 (0.6–3.99)	0.4	–	–
Anti‐PL‐12	1.23 (0.47–3.16)	0.7	–	–
Anti‐EJ	1.65 (0.44–6.24)	0.4	–	–
Anti‐Ro52 (TRIM21)	0.64 (0.25–1.62)	0.3	–	–
**Laboratory indicators at ILD diagnosis**				
C‐reactive protein	**1.01 (1.01–1.02)**	**<0.001**	1.01 (0.98–1.04)	0.67
Ferritin	1.72 (0.41–7.94)	0.5	–	–
Creatine phosphokinase	**0.38 (0.22–0.63)**	**<0.001**	0.25 (0.06–1.02)	0.05
**ILD outcomes at ILD diagnosis**				
UIP pattern	0.54 (0.08–2.57)	0.5	–	–
NSIP pattern	0.5 (0.15–1.66)	0.3	–	–
OP pattern	**3.78 (1.02–15.9)**	**0.047**	**66.8 (3.39–1316)**	**0.006**
Pleural effusion	**10.7 (3.62–39.3)**	**<0.001**	**20.2 (1.09–373)**	**0.04**

*Note*: Bold font indicates statistically significant results (*p* < 0.05).

Abbreviations: aOR, adjusted odds ratio; ASyS, antisynthetase syndrome; BMI, body mass index; CI, confidence interval; ILD, interstitial lung disease; NSIP, nonspecific interstitial pneumonia; OP, organized pneumonia; OR, odds ratio; RP‐ILD, rapidly progressive interstitial lung disease; UIP, usual interstitial pneumonia.

### ILD outcomes during follow‐up

The median follow‐up time was 49 (24–86) months (Table 1). Seventy‐one (54%) patients experienced at least one ILD relapse during follow‐up. Patients with RP‐ILD at ILD diagnosis had more instances of chronic respiratory failure and more frequently received long‐term oxygen therapy during follow‐up (Table 1). The proportion of patients with at least one ILD relapse during the follow‐up was not different between patients with RP‐ILD at diagnosis and patients without RP‐ILD at diagnosis (59% vs. 49%, respectively, *p* = 0.28).

Overall or transplant‐free survival after 5 years of follow‐up from the time of ILD diagnosis tended to be worse in patients with RP‐ILD at diagnosis (Fig. S2). When overall or transplant‐free survival from the time of ILD diagnosis was evaluated according to anti‐ARS antibodies, patients with anti‐PL‐7 antibodies tended to have the worst prognosis among all groups after 5 years of follow‐up (*p* = 0.062).

According to the multivariate analysis, only cardiac involvement (pericarditis or myocarditis) at time of ASyS diagnosis was significantly associated with mortality in the Cox regression model (Table S4).

### Unsupervised clustering

Unsupervised hierarchical clustering was used to separate patients into four distinct clusters (Fig. S1). Comparative tables for each cluster were included in the supplementary materials (Tables S5–S10).

Cluster 1 (*n* = 62, “systemic cluster”) almost exclusively included patients with anti‐Jo‐1 antibodies (92%). These patients had more systemic presentations, including mechanic's hand (45%), microvascular involvement (47%), joint involvement (79%), and muscle involvement (65%), but fewer instances of fever and cardiac involvement than patients in the other clusters (Table 3). Compared with patients in the other clusters, patients in Cluster 1 had the best pulmonary prognosis, with fewer cases of RP‐ILD at diagnosis (18%), chronic respiratory failure, and death and/or lung transplantation during follow‐up.

**Table 3:** *Comparison of clusters derived from hierarchical clustering analysis in antisynthetase syndrome (ASyS)‐interstitial lung disease (ILD) patients*.

Variable	Cluster 1 (*N* = 62)	Cluster 2 (*N* = 40)	Cluster 3 (*N* = 20)	Cluster 4 (*N* = 10)
**Demographics**				
Female sex, *n/N* (%)	**44/62 (71)** ^a^, ^b^	**18/40 (45)** ^d^, ^e^	**20/20 (100)**	**9/10 (90)**
Age, *n/N* (%)				
≤40 years	**9/62 (15)** ^a^	**0/40 (0)** ^d^, ^e^	**6/20 (30)**	**0/10 (0)**
40–60 years	**44/62 (71)** ^a^	**8/40 (20)** ^d^, ^e^	**9/20 (45)**	**6/10 (60)**
>60 years	**9/62 (15)** ^a^	**32/40 (80)** ^d^, ^e^	**5/20 (25)**	**4/10 (40)**
Comorbidities				
Cancer‐associated myositis, *n/N* (%)	**4/62 (7)** ^a^	**10/40 (25)**	2/20 (10)	2/10 (20)
Overlapping CTD, *n/N* (%)	**14/62 (23)** ^a^	**2/40 (5)** ^d^	**6/20 (30)**	2/10 (20)
**Clinical manifestations at ASyS diagnosis**				
Skin involvement, *n/N* (%)				
Mechanic's hands	**28/62 (45)** ^a^	**5/40 (13)**	6/20 (30)	2/10 (20)
Typical dermatomyositis signs	11/62 (18)	4/40 (10)	6/20 (30)	0/10 (0)
Microvascular involvement, *n/N* (%)	**29/62 (47)** ^a^	**9/40 (23)**	7/20 (35)	2/10 (20)
Joint involvement, *n/N* (%)	**49/62 (79)** ^a^, ^b^, ^c^	**17/40 (43)**	**10/20 (50)**	**2/10 (20)**
Muscle involvement, *n/N* (%)	**40/62 (65)** ^a^, ^b^	**18/40 (45)**	**6/20 (30)**	3/10 (30)
Severe muscle involvement	**24/62 (39)** ^a^, ^b^	**7/40 (18)**	**2/20 (10)**	3/10 (30)
Fever, *n/N* (%)	**12/61 (20)** ^a^, ^h^	**20/40 (50)**	8/20 (40)	4/10 (40)
Pericarditis and/or myocarditis, *n/N* (%)	**3/61 (5)** ^a^, ^h^	**7/40 (18)**	2/20 (10)	0/10 (0)
**Antibodies at ASyS diagnosis**				
Anti‐Jo‐1, *n/N* (%)	**57/62 (92)** ^a^, ^b^, ^c^	**23/40 (58)** ^d^, ^e^	**0/20 (0)**	**0/10 (0)**
Anti‐PL‐7, *n/N* (%)	**5/62 (8)** ^a^	**16/40 (40)** ^d^, ^e^	**0/20 (0)**	**0/10 (0)**
Anti‐PL‐12, *n/N* (%)	**0/62 (0)** ^b^	**1/40 (3)** ^d^	**20/20 (100)** ^f^	0/10 (0)
Anti‐EJ, *n/N* (%)	**0/62 (0)** ^c^	**0/40 (0)** ^e^	**0/20 (0)** ^f^	**10/10 (100)**
Anti‐Ro52 (TRIM21), *n/N* (%)	17/34 (50)^h^	10/20 (50)^i^	9/13 (69)^j^	4/8 (50)^k^
**Laboratory indicators at ILD diagnosis**				
Creatine phosphokinase, *n/N* (%)				
≤170 IU/L	**12/62 (19)** ^a^	**17/40 (43)**	9/20 (45)	5/10 (50)
170–800 IU/L	**16/62 (26)** ^a^	**7/40 (18)**	5/20 (25)	1/10 (10)
800–2300 IU/L	**14/62 (23)** ^a^	**13/40 (33)**	4/20 (20)	3/10 (30)
2300–7000 IU/L	**17/62 (27)** ^a^	**3/40 (8)**	2/20 (10)	1/10 (10)
>7000 IU/L	**3/62 (5)** ^a^	**0/40 (0)**	0/20 (0)	0/10 (0)
**ILD outcomes at ILD diagnosis**				
Pleural effusion, *n/N* (%)	**1/55 (2)** ^a^, ^b^, ^h^	**16/38 (42)** ^i^	**4/20 (20)**	1/10 (10)
FVC <70% predicted, *n/N* (%)	23/62 (37)	20/40 (50)	10/20 (50)	7/10 (70)
FVC, % predicted^g^	**79 (65–88)** ^c^	65 (54–84)	69 (61–77)	**50 (45–72)**
DL_CO_, % predicted^g^	**58 (44–71)** ^a^	**45 (32–52)**	48 (41–56)	47 (45–53)
RP ILD, *n/N* (%)	**11/62 (18)** ^a^, ^c^	**27/40 (68)** ^d^	**8/20 (40)**	**5/10 (50)**
**ILD outcomes during follow‐up**				
Number of ILD relapses^g^	**0 (** **0–1)** ^a^	**1 (0–2)**	2 (0–3)	1 (0–1)
Suspected or confirmed PH, *n/N* (%)	8/62 (13)	11/40 (28)	5/20 (25)	3/10 (30)
Chronic respiratory failure, *n/N* (%)	**3/62 (5)** ^a^, ^b^, ^c^	**15/39 (38)** ^i^	**7/20 (35)**	**4/10 (40)**
Death and/or lung transplantation, *n/N* (%)	**2/62 (3)** ^a^	**22/40 (55)** ^d^, ^e^	**3/20 (15)**	**1/10 (10)**
**Number of treatments used during follow‐up** ^g^	3 (2–4)	3 (2–4)	3 (2–4)	3 (2–3)

*Note*: Alphabetical superscripts of clusters indicate statistical differences among subgroups using the Wilcoxon rank sum test (continuous variables) and *χ* ^2^ test or Fisher's exact test (categorical variables). Bold font indicates statistically significant results (*p* < 0.05).

Abbreviations: ASyS, antisynthetase syndrome; CTD, connective tissue disease; DLCO, diffusion capacity of the lung for carbon monoxide; FVC, forced vital capacity; ILD, interstitial lung disease; IQR, interquartile range; PH, pulmonary hypertension; RP ILD, rapidly progressive interstitial lung disease.

^a^ Presents statistical differences between Clusters 1 and 2.

^b^ Presents statistical differences between Clusters 1 and 3.

^c^ Presents statistical differences between Clusters 1 and 4.

^d^ Presents statistical differences between Clusters 2 and 3.

^e^ Presents statistical differences between Clusters 2 and 4.

^f^ Presents statistical differences between Clusters 3 and 4.

^g^ Data are expressed as the median and interquartile range (IQR).

^h^ *N* ≠ 62 due to missing data.

^i^ *N* ≠ 40 due to missing data.

^j^ *N* ≠ 20 because of missing data.

^k^ *N* ≠ 10 because of missing data.

Patients in Cluster 2 (*n* = 40, “older age cluster”) were more likely to be old and male, have anti‐Jo‐1 or anti‐PL‐7 antibodies (in almost equal parts), and have more associated cancers (25%) (Table 3). Half of the patients had fever, 42% had pleural effusion, and 18% had cardiac manifestations (mainly pericarditis). With respect to ILD prognosis, 68% of patients had RP‐ILD at diagnosis, 38% had chronic respiratory failure during follow‐up, and 55% died.

Cluster 3 (*n* = 20, “anti‐PL‐12 cluster”) was exclusively represented by patients with anti‐PL‐12 antibodies (Table 3). The main extra‐respiratory manifestations were joint involvement (50%) and microvascular involvement (35%). RP‐ILD at diagnosis was reported in 40% of patients. During follow‐up, the median number of ILD relapses was 2 (0−3), 35% of patients developed chronic respiratory failure, and 15% died.

Cluster 4 (*n* = 10, “anti‐EJ cluster”) was exclusively represented by patients with anti‐EJ antibodies (Table 3). At diagnosis, 50% of patients had RP‐ILD. During follow‐up, 40% of the patients developed chronic respiratory failure, and one patient died.

## Discussion

Our study evaluated factors associated with RP‐ILD at the time of ILD diagnosis in a large multicenter ASyS‐ILD cohort, with comprehensive data collection enabling multivariate analysis.

Multivariate analysis identified fever and lower CPK level as independently associated with RP‐ILD, aligning with previous reports in patients with IIM [31, 32, 33]. To the best of our knowledge, this is the first study to specifically identify independent factors associated with RP‐ILD in ASyS using multivariate logistic regression analysis. Previous studies have been limited to descriptive comparisons of ASyS patients with versus without RP‐ILD, or analyses within broader IIM‐ILD populations not restricted to ASyS [17, 32]. Some studies have also compared RP‐ILD rates across autoantibody subgroups [17, 20]. However, none have employed a multivariate model focused solely on ASyS to disentangle the effects of various clinical, laboratory, imaging, and serologic variables. We excluded dyspnea scores, PFTs, and blood gas results, as they are part of the definition of RP‐ILD and are related to pulmonary severity (risk of multicollinearity). Pleural effusion was present in nearly all RP‐ILD patients and independently associated with RP‐ILD at diagnosis. A prior Chinese study also found an association between pleural effusion and RP‐ILD in IIM [31]. Although this should be interpreted with caution due to the high proportion of missing data, OP pattern was also associated with RP‐ILD at diagnosis in our cohort. The OP pattern is typically linked to acute inflammatory forms of ILD and has been reported more frequently among RP‐ILD patients in a cohort of IIM‐ILD patients that included approximately one‐third ASyS patients [18, 32]. Fever, pleural effusion, and OP pattern may reflect the high degree of inflammation in RP‐ILD patients, as previously reported in cohorts of ASyS patients and in anti‐MDA5 positive patients [34, 35, 36]. Indeed, a high level of lung inflammation may be a primary determinant of ILD severity at time of diagnosis, as evidenced by the frequent clinical improvement following the initiation of corticosteroids and immunosuppressive treatments. Notably, we identified a novel association between male sex and RP‐ILD in ASyS patients, a finding not previously described in IIM [37, 38]. Male sex has been associated with progressive ILD in other CTD such as systemic sclerosis and mixed CTD. Liu et al. also reported a higher proportion of male patients with ILD CT findings deterioration during follow‐up in ASyS [39]. This observation warrants confirmation in future studies.

We found no strong association between anti‐ARS antibodies and RP‐ILD at diagnosis, but trends suggested less severe ILD in anti‐Jo‐1‐positive patients (higher FVC, lower dyspnea) and more severe ILD in other anti‐ARS subgroups as described in previous studies [11, 15]. Anti‐Ro52 (anti‐TRIM21) antibodies may be associated with ILD prevalence and severity, particularly in ASyS [40]. In our cohort, however, anti‐Ro52 antibodies were not associated with RP‐ILD at diagnosis but data were missing in 57 patients. Given the ongoing debate regarding their clinical significance, some laboratories opt not to report anti‐Ro52 (anti‐TRIM21) antibodies to avoid potential confusion or misinterpretation. A broader spectrum of autoantibodies—such as rheumatoid factor, ACPA, and anti‐dsDNA—were not collected, as they are not systematically tested in patients in the absence of suspicion for overlapping CTD.

Our primary goal was to assess RP‐ILD determinants at diagnosis, given its prognostic relevance. Given the heterogeneity of the disease, we applied an unsupervised approach (hierarchical clustering) using over 20 variables to uncover distinct phenotypes among ASyS‐ILD patients, without making prior assumptions about RP‐ILD status or autoantibody profiles. This strategy provides more nuanced insights than simply comparing patients with versus without RP‐ILD or grouping them by autoantibody profile, as it enables the identification of subgroups that may not align with predefined categories but may reveal clinically meaningful patterns. We identified two clinically relevant clusters concerning ILD outcomes and prognosis, which could enhance patient stratification in ASyS‐ILD at initial diagnosis. Patients in Cluster 1 (systemic cluster) had the best prognosis, systemic symptoms, and predominantly anti‐Jo‐1 antibodies, supporting several similar descriptions in the literature [11, 30]. Hervier et al. identified a similar cluster with systemic features and anti‐Jo‐1 antibodies, whereas a second cluster comprised patients with isolated ILD and anti‐PL‐7/PL‐12 antibodies. Cluster 2 (older age cluster) had higher RP‐ILD prevalence at ILD diagnosis, chronic respiratory failure, and mortality, and showed an inflammatory phenotype (fever, pleuritis, pericarditis). A similar inflammatory phenotype with severe prognosis was described in Asian cohorts [33, 41]. A Chinese cohort of 701 ASyS patients identified an RP‐ILD phenotype with older age, fever, high CRP level, and worse survival through unsupervised clustering analysis, independent of antibody type [22]. Transcriptomic data in that cluster revealed enrichment of coagulation, platelet activation, and inflammatory genes. Interpretation of Clusters 3 and 4 was limited due to the small number of patients in these clusters. The weak average silhouette width suggests the relative quality of our clustering analysis. Nonetheless, cluster characteristics support our results from the multivariate regression analysis and align with previous cohorts utilizing unsupervised clustering analysis.

RP‐ILD at ILD diagnosis was frequent in our cohort (∼40%), mainly due to respiratory failure with hypoxemia. Variability in RP‐ILD prevalence across studies likely reflects differing definitions [17, 22, 30]. In our cohort, the higher prevalence of RP‐ILD may be attributed to our broad definition—encompassing both respiratory failure‐related forms of ILD and worsening ILD in the initial months—which was employed to thoroughly capture patients exhibiting this phenotype. Our definition of respiratory failure may have inadvertently encompassed a few patients receiving oxygen therapy but without significant hypoxemia. It is less likely but still possible that we included patients with severe ILD whose respiratory failure persisted beyond 3 months. A new definition of RP‐ILD was recently proposed in the 2023 ACR/CHEST guidelines, which could aid in standardizing future research in this field [21]. At the time of diagnosis, patients with RP‐ILD predominantly exhibited moderate FVC and DLC_O_ values. One possible explanation is that PFTs were not performed in the more severe cases (FVC and DLC_O_ values were missing in 20 and 24 patients with RP‐ILD at time of diagnosis, respectively). Patients with RP‐ILD had similar baseline FVC and DLC_O_ values in a large Chinese cohort of ASyS patients [22].

Patient demographics, clinical manifestations, and antibody distributions were consistent with prior large European cohorts [12, 42, 43]. However, no patients with anti‐OJ antibodies were included in our study. Commercial immunoassays kits used in most centers are not able to detect these rare antibodies [44].

In our cohort, 20% died during follow‐up. Cause of death was often undocumented, so ILD‐specific mortality could not be determined. We observed a trend toward shorter survival in RP‐ILD patients at diagnosis and those with anti‐PL‐7 antibodies, as previously reported [42, 45, 46]. Despite less chronic respiratory failure (14%), anti‐PL‐7 patients had the worst survival, possibly due to older age (median 64 vs. 55 years). Age tended to be associated with mortality in multivariate Cox analysis, consistent with previous studies [11, 46, 47]. Cox regression analysis was constrained by the low number of events, which may have affected the robustness of the findings.

Our study had several limitations, mainly due to its retrospective design and the rarity of ASyS. We chose to exclude the few patients with rare or double anti‐ARS antibodies to ensure a more homogeneous ASyS population. To properly assess the main outcome, we excluded several eligible patients due to missing data at ILD diagnosis, which may also have introduced selection bias. Comorbidities like chronic obstructive pulmonary disease or cardiovascular diseases were not evaluated. A delay in ASyS diagnosis, especially in those with isolated ILD, might also represent a potential risk factor for RP‐ILD at diagnosis [20]. Unfortunately, determination of the first symptom date was missing in most patients in our cohort, and an evaluation of diagnostic delay was not feasible. Furthermore, the date of ILD diagnosis was not recorded when ILD was identified prior to ASyS diagnosis. A prolonged evolution of ILD prior to ASyS diagnosis may have led to delayed initiation of appropriate treatment, potentially resulting in worse ILD outcomes. Ethnicity was not analyzed due to the region's predominantly Caucasian population. Antibody titers were not assessed due to varying testing methods across centers. Finally, the impact of autoantibodies other than anti‐ARS and anti‐Ro52 on the risk of RP‐ILD was not assessed.

## Conclusion

Fever, pleural effusion, and OP pattern were independently associated with RP‐ILD at diagnosis, whereas anti‐ARS autoantibodies were not sufficient to account for the presence of RP‐ILD at diagnosis. We confirmed distinct clusters of ASyS‐ILD patients through unsupervised analysis in a large French cohort, including a systemic phenotype linked to non‐RP ILD and anti‐Jo‐1 antibodies, and an inflammatory phenotype associated with older age, RP‐ILD, and a worse prognosis. Although anti‐ARS antibodies may be important for phenotyping ASyS patients, fever, pleural effusion, and OP pattern represent key inflammatory biomarkers that could help clinicians more accurately assess RP‐ILD in these patients. Closer monitoring and/or more aggressive treatment during the initial phase in at‐risk patients could pose a future challenge in the management of ASyS‐ILD patients to prevent the development of RP‐ILD.

## Conflict of interest statement

The authors declare no conflicts of interest.

## Funding information

No specific funding was received from any bodies in the public, commercial, or not‐for‐profit sectors to carry out the work described in this article.

## Supporting information




**Table S1**: Variables included in the hierarchical clustering analysis.
**Table S2**: Characteristics of patients with and without associated CTD.
**Table S3**: Patient characteristics according to the anti‐aminoacyl‐tRNA‐synthetase antibodies.
**Table S4**: Factors associated with all‐cause mortality according to Cox regression analysis (after selection of clinically pertinent variables).
**Table S5**: Comparison of clusters 1 and 2 derived from hierarchical clustering analysis in ASyS‐ILD patients.
**Table S6**: Comparison of clusters 1 and 3 derived from hierarchical clustering analysis in ASyS‐ILD patients.
**Table S7**: Comparison of clusters 1 and 4 derived from hierarchical clustering analysis in ASyS‐ILD patients.
**Table S8**: Comparison of clusters 2 and 3 derived from hierarchical clustering analysis in ASyS‐ILD patients.
**Table S9**: Comparison of clusters 2 and 4 derived from hierarchical clustering analysis in ASyS‐ILD patients.
**Table S10**: Comparison of clusters 3 and 4 derived from hierarchical clustering analysis in ASyS‐ILD patients.

**Figure S1**: Hierarchical clustering analysis of ASyS‐ILD patients according to included variables at baseline and during follow‐up (cumulative data). (A) Multiple correspondence analysis factor map. Factor map showing the row data (patients) used to generate the dendrogram. Dimensions 1 and 2 cumulatively explained 20.9% of the total variance. (B) Dendrogram. The *y*‐axis indicates the height of fusion into the clusters proposed, and the *x*‐axis indicates ASyS‐ILD patients (*n* = 132). A hierarchical tree indicates ASyS‐ILD patients according to the cluster to which they belong. Alt text: Visual representation of the four clusters derived from unsupervised analysis (panel A displays a factor map, and panel B presents a dendrogram). ASyS, antisynthetase syndrome; ILD, interstitial lung disease.

**Figure S2**: Overall survival or transplant‐free survival from the time of ILD diagnosis according to rapidly progressive ILD at time of ILD diagnosis (A) and anti‐ARS antibodies (B). Alt text: Kaplan–Meier survival curves comparing patients with rapidly progressive interstitial lung disease (RP‐ILD) versus those without (panel A) and patients stratified by anti‐aminoacyl‐tRNA synthetase antibody status (panel B), indicating a trend toward worse survival in RP‐ILD patients and those positive for anti‐PL‐7 antibodies.

## Data Availability

The data underlying this article will be shared upon reasonable request to the corresponding author.
