# Supplementary material for: Clinical profiles associated with rapidly progressive interstitial lung disease in antisynthetase syndrome: A multicentric cohort study (TYPASS study)
Source: J Intern Med. 2025 Dec 10;299(3):365–80. doi: 10.1111/joim.70058 (PMC12869006; doi:10.1111/joim.70058)
Supplement: Supplementary file 1 — Table S1: Variables included in the hierarchical clustering analysis. Table S2: Characteristics of patients with and without associated CTD. Table S3: Patient characteristics according to the anti‐aminoacyl‐tRNA‐synthetase antibodies. Table S4: Factors associated with all‐cause mortality according to Cox regression analysis (after selection of clinically pertinent variables). Table S5: Comparison of clusters 1 and 2 derived from hierarchical clustering analysis in ASyS‐ILD patients. Table S6: Comparison of clusters 1 and 3 derived from hierarchical clustering analysis in ASyS‐ILD patients. Table S7: Comparison of clusters 1 and 4 derived from hierarchical clustering analysis in ASyS‐ILD patients. Table S8: Comparison of clusters 2 and 3 derived from hierarchical clustering analysis in ASyS‐ILD patients. Table S9: Comparison of clusters 2 and 4 derived from hierarchical clustering analysis in ASyS‐ILD patients. Table S10: Comparison of clusters 3 and 4 derived from hierarchical clustering analysis in ASyS‐ILD patients. [file JOIM-299-365-s002.docx]

**Supplementary data**

**Figure Legends**

**Supplementary Figure S1**. Hierarchical clustering analysis of ASyS-ILD patients according to included variables at baseline and during follow-up (cumulative data). A) Multiple correspondence analysis factor map. Factor map showing the row data (patients) used to generate the dendrogram. Dimensions 1 and 2 cumulatively explained 20.9% of the total variance. B) Dendrogram. The y-axis indicates the height of fusion into the clusters proposed, and the x-axis indicates ASyS-ILD patients (n = 132). A hierarchical tree indicates ASyS-ILD patients according to the cluster to which they belong. ASyS: antisynthetase syndrome; ILD: interstitial lung disease.

Alt text: Visual representation of the four clusters derived from unsupervised analysis (panel A displays a factor map and panel B presents a dendogram).

**Supplementary Figure S2**. Overall survival or transplant-free survival from the time of ILD diagnosis according to rapidly-progressive ILD at time of ILD diagnosis (A) and anti-ARS antibodies (B). ILD: interstitial lung disease; Anti-ARS antibodies: anti-aminoacyl-tRNA-synthetase antibodies.

Alt text: Kaplan-Meier survival curves comparing patients with rapidly-progressive interstitial lung disease (RP-ILD) versus those without (panel A), and patients stratified by anti-aminoacyl-tRNA synthetase antibody status (panel B), indicating a trend toward worse survival in RP-ILD patients and those positive for anti-PL-7 antibodies.

**Tables**

**Supplementary Table S1.** Variables included in the hierarchical clustering analysis

| **Variable** | |
| --- | --- |
| Sex | Anti-ARS antibodies (anti-Jo-1, anti-PL-7, anti-PL-12, anti-OJ, anti-EJ) |
| Age | CK level |
| Cancer-associated myositis | Anti-Ro52 antibodies |
| Mechanic’s hand | Pleural effusion |
| Typical dermatomyositis signs | FVC < 70% of the predicted value |
| Microvascular manifestations | RP-ILD at time of ILD diagnosis |
| Joint involvement | ILD relapse |
| Muscle involvement | Chronic respiratory failure |
| Severe muscle involvement | Death and/or lung transplantation |
| Cardiac manifestations | Number of treatments received |
| Fever |  |

ARS: anti-aminoacyl-tRNA-synthetase; CK: creatine kinase; FVC: forced vital capacity; ILD: interstitial lung disease, RP ILD: rapidly progressive interstitial lung disease.

**Supplementary Table S2.** Characteristics of patients with and without associated CTD

| **Variable** | | | | **Non-associated CTD**  **(*N* = 108)** | **Associated**  **CTD**  **(*N* = 24)** | **P value** |
| --- | --- | --- | --- | --- | --- | --- |
| **Demographics** | | | |  |  |  |
| Female, *n (%)* | | | | **70/108 (65)** | **21/24 (88)** | **0.03** |
| Age, years^a^ | | | | **56 (47─67)** | **53 (37─57)** | **0.03** |
| BMI, kg/m²^a^ | | | | 27 (24─30) | 26 (23─32) | > 0.9 |
| Former or current smokers, *n (%)* | | | | 53/99 (54)^‡^ | 17/21 (71)* | 0.13 |
| Cancer-associated myositis, *n (%)* | | | | 16/108 (15) | 2/24 (8) | 0.5 |
| **Clinical manifestations at ASyS diagnosis** | | | |  |  |  |
| Skin involvement, *n (%)* | | | | 41/108 (38) | 10/24 (42) | 0.7 |
|  | | Mechanic’s hands | | 33/108 (35) | 8/24 (33) | 0.8 |
|  | | Typical dermatomyositis signs | | 18/108 (17) | 3/24 (13) | 0.8 |
| Microvascular involvement, *n (%)* | | | | 37/108 (34) | 10/24 (42) | 0.5 |
|  | | Raynaud’s phenomenon | | 26/108 (24) | 8/24 (33) | 0.3 |
|  | | Nailfold capillaroscopy abnormalities | | 14/37 (38)^‡^ | 4/9 (44)* | 0.7 |
| Joint involvement, *n (%)* | | | | 61/108 (56) | 17/24 (71) | 0.2 |
|  | | Arthralgia | | 61/108 (56) | 16/24 (67) | 0.4 |
|  | | Synovitis | | 29/108 (27) | 11/24 (46) | 0.07 |
|  | | Erosions (radiography) | | 1/21 (5)^‡^ | 2/12 (17)* | 0.5 |
| Muscle involvement, *n (%)* | | | | 56/108 (52) | 11/24 (46) | 0.6 |
|  | | Muscle weakness (MRC ≤ 3) | | 19/106 (18)^‡^ | 5/24(21)* | 0.8 |
|  | | Dysphagia | | 3/108 (3) | 2/24 (8) | 0.2 |
|  | | Severe muscle involvement | | 29/108 (27) | 7/24 (29) | 0.8 |
| Fever, *n (%)* | | | | 36/107 (34)^‡^ | 8/24 (33) | > 0.9 |
| Pericarditis, *n (%)* | | | | 9/107 (8)^‡^ | 3/24 (13) | 0.5 |
| Myocarditis, *n (%)* | | | | 2/107 (2)^‡^ | 0/24 (0) | > 0.9 |
| **Antibodies at ASyS diagnosis** | | | |  |  |  |
| Anti-Jo-1, *n (%)* | | | | 65/108 (60) | 15/24 (63) | 0.8 |
| Anti-PL-7, *n (%)* | | | | 20/108 (19) | 1/24 (4) | 0.12 |
| Anti-PL-12, *n (%)* | | | | 15/108 (14) | 6/24 (25) | 0.2 |
| Anti-EJ, *n (%)* | | | | 8/108 (7) | 2/24 (8) | > 0.9 |
| Anti-Ro52 (TRIM21), *n (%)* | | | | 29/59 (49)^‡^ | 11/16 (69)* | 0.2 |
| **Laboratory indicators at ILD diagnosis** | | | |  |  |  |
| C-reactive protein, mg/L^a^ | | | | 24 (13─58) | 25 (7─51) | 0.7 |
| Ferritin, µg/L^a^ | | | | 304 (86─574) | 415 (138─545) | 0.8 |
| Creatine phosphokinase, IU/L^a^ | | | | 687 (111─2183) | 252 (122─2723) | 0.8 |
| **ILD outcomes at ILD diagnosis** | | | |  |  |  |
| Dyspnea on mMRC scale, *n (%)* | | | |  |  | 0.9 |
| 0 | | | | 16/85 (19)^‡^ | 3/19 (16)* |  |
| 1 | | | | 11/85 (13)^‡^ | 4/19 (21)* |  |
| 2 | | | | 18/85 (21)^‡^ | 5/19 (26)* |  |
| 3 | | | | 17/85 (20)^‡^ | 3/19 (16)* |  |
| 4 | | | | 23/85 (27)^‡^ | 4/19 (21)* |  |
| Cough, *n (%)* | | | | 62/103 (60)^‡^ | 13/24 (54)* | 0.6 |
| ILD pattern, *n (%)* | | | |  |  |  |
| UIP | | |  | 6/57 (11)^‡^ | 2/14 (14)* | 0.7 |
| NSIP | | |  | 47/57 (82)^‡^ | 10/14 (71)* | 0.5 |
| OP | | |  | 10/57 (18)^‡^ | 1/14 (7)* | 0.7 |
| Pleural effusion, *n (%)* | | | | 18/101 (18)^‡^ | 4/22 (18)* | > 0.9 |
| Pulmonary function test results | | | |  |  |  |
|  | PaO_2_, mmHg^a^ | | | 70 (63─90) | 76 (68─89) | 0.2 |
|  | FVC, % predicted^a^ | | | 72 (57─85) | 74 (59─80) | > 0.9 |
|  | TLC, % predicted^a^ | | | 73 (59─88) | 77 (59─101) | 0.4 |
|  | DL_CO_, % predicted^a^ | | | 50 (41─61) | 63 (43─86) | 0.1 |
|  | DL_CO_/VA, % predicted^a^ | | | 73 (64─85) | 85 (50─94) | 0.8 |
| Rapidly progressive ILD, *n (%)* | | | | 45/108 (42) | 6/24 (25) | 0.13 |
|  | Respiratory failure, *n (%)* | | | 41/108 (38) | 5/24 (21) | 0.11 |
|  | Worsening ILD in the first 3 months, *n (%)* | | | 4/108 (4) | 1/24 (4) | > 0.9 |
| **ILD outcomes during follow-up** | | | |  |  |  |
| Follow-up time, months^a^ | | | | 43 (19─85) | 63 (38─86) | 0.2 |
| Number of ILD relapses^a^ | | | | 1 (0─2) | 0 (0─2) | 0.4 |
| Confirmed pulmonary hypertension, *n (%)* | | | | 10/108 (9) | 1/24 (4) | 0.7 |
| Chronic respiratory failure, *n (%)* | | | | 25/107(23)^‡^ | 4/24 (17) | 0.5 |
| Long-term oxygen therapy, *n (%)* | | | | 23/106 (22)^‡^ | 4/24 (17) | 0.8 |
| Lung transplantation, *n (%)* | | | | 1/108 (1) | 0/24 (0) | > 0.9 |
| Death, *n (%)* | | | | 24/108 (22) | 3/24 (13) | 0.4 |
| **Treatments used during follow-up** | | | |  |  |  |
| Number of treatments used during follow-up^a^ | | | | 3 (2─4) | 3 (2─4) | 0.7 |
| Corticosteroids, *n (%)* | | | | 103/108 (95) | 22/24 (92) | 0.6 |
| Hydroxychloroquine, *n (%)* | | | | **6/108 (6)** | **7/24 (29)** | **0.002** |
| Methotrexate, *n (%)* | | | | 42/108 (39) | 12/24 (50) | 0.3 |
| Azathioprine, *n (%)* | | | | 30/108 (28) | 5/24 (21) | 0.5 |
| Cyclophosphamide, *n (%)* | | | | 32/108 (30) | 4/24 (17) | 0.2 |
| Mycophenolate mofetil, *n (%)* | | | | 47/108 (44) | 7/24 (29) | 0.2 |
| Rituximab, *n (%)* | | | | 39/108 (36) | 11/24 (46) | 0.4 |
| CNI, *n (%)* | | | | 12/108 (11) | 0/24 (0) | 0.12 |
| IVIg, *n (%)* | | | | 20/108 (19) | 4/24 (17) | > 0.9 |
| Antifibrotic drugs, *n (%)* | | | | 3/108 (3) | 2/24 (8) | 0.2 |

Bold font indicates statistically significant results (P < 0.05). ^a^ Data are expressed as medians with interquartile ranges (IQRs). ^‡^ N ≠ XX due to missing data. * N ≠ XX due to missing data. ASyS: antisynthetase syndrome; BMI: body mass index; CNI: calcineurin inhibitor; CTD: connective tissue disease; DL_CO_: diffusion capacity of the lung for carbon monoxide; DL_CO_/VA: diffusion capacity of the lung for carbon monoxide per lung volume; FVC: forced vital capacity; ILD: interstitial lung disease; IVIg: intravenous immunoglobulin; mMRC: modified Medical Research Council dyspnea scale; MRC: Medical Research Council muscle testing scale; NSIP: nonspecific interstitial pneumonia; OP: organized pneumonia; PaO2: arterial partial pressure of oxygen; TLC: total lung capacity; TRV: tricuspid regurgitation velocity; UIP: usual interstitial pneumonia.

**Supplementary Table S3.** Patient characteristics according to the anti-aminoacyl-tRNA-synthetase antibodies

| **Variable** | | **Anti-Jo-1**  **(*N* = 80)** | **Anti-PL-7**  **(*N* = 21)** | **Anti-PL-12**  **(*N* = 21)** | **Anti-EJ**  **(*N* = 10)** | **Non-anti-Jo-1**  **(*N* = 52)** | **P value^ɪ^** |
| --- | --- | --- | --- | --- | --- | --- | --- |
| **Demographics** | |  |  |  |  |  |  |
| Female sex, *n/N (%)* | | **50/80 (63)** | 12/21 (57) | 20/21 (95) | 9/10 (90) | **41/52 (79)** | **0.047** |
| Age, years^†^ | | 54 (47─64) | 64 (52─70) | 56 (34─64) | 56 (54─74) | 57 (47─67) | 0.4 |
| BMI, kg/m²^†^ | | 27 (24─30) | 26 (24─29) | 26 (22─31) | 29 (25─32) | 26 (24─31) | 0.6 |
| Former or current smokers, *n/N (%)* | | 37/72 (51) ^‡^ | 14/20 (70)* | 12/20 (60)* | 5/8 (63)^ˠ^ | 31/48 (65)^ɫ^ | 0.2 |
| Comorbidities, *n/N (%)* | |  |  |  |  |  |  |
| Cancer-associated myositis | | 10/80 (13) | 3/21 (14) | 3/21 (14) | 2/10 (20) | 8/52 (15) | 0.6 |
| Overlapping connective tissue disease | | 15/80 (19) | 1/21 (5) | 6/21 (29) | 2/10 (20) | 9/52 (17) | 0.8 |
|  | Systemic lupus | 2/80 (3) | 0/21 (0) | 1/21 (5) | 1/10 (10) | 2/52 (4) | 0.6 |
|  | Gougerot-Sjögren syndrome | 12/80 (15) | 0/21 (0) | 3/21 (14) | 1/10 (10) | 4/52 (8) | 0.2 |
|  | Systemic sclerosis | 1/80 (1) | 0/21 (0) | 0/21 (0) | 0/10 (0) | 0/52 (0) | > 0.9 |
|  | Rheumatoid arthritis | 3/80 (4) | 1/21 (5) | 3/21 (14) | 0/10 (0) | 4/52 (8) | 0.4 |
| **Clinical manifestations at ASyS diagnosis** | |  |  |  |  |  |  |
| Skin involvement, *n/N (%)* | | 30/80 (38) | 7/21 (33) | 12/21 (57) | 2/10 (20) | 21/52 (40) | 0.7 |
|  | Mechanic’s hands | 27/80 (34) | 6/21 (29) | 6/21 (29) | 2/10 (20) | 14/52 (27) | 0.4 |
|  | Typical dermatomyositis signs | 10/80 (13) | 4/21 (19) | 7/21 (33) | 0/10 (0) | 11/52 (21) | 0.2 |
| Microvascular involvement, *n/N (%)* | | 32/80 (40) | 6/21 (29) | 7/21 (33) | 2/10 (20) | 15/52 (29) | 0.2 |
|  | Raynaud’s phenomenon | 23/80 (29) | 4/21 (19) | 5/21 (24) | 2/10 (20) | 11/52 (21) | 0.3 |
|  | Nailfold capillaroscopy abnormalities | 23/80 (29) | 2/7 (29)* | 6/8 (75)* | 0/2 (0)^ˠ^ | 8/17 (47)^ɫ^ | 0.4 |
| Joint involvement, *n/N (%)* | | **62/80 (78)** | 4/21 (19) | 10/21 (48) | 2/10 (20) | **16/52 (31)** | **< 0.001** |
|  | Arthralgia | **61/80 (76)** | 4/21 (19) | 10/21 (48) | 2/10 (20) | **16/52 (31)** | **< 0.001** |
|  | Synovitis | **37/79 (47)^‡^** | 0/21 (0) | 2/21 (10) | 1/10 (10) | **3/52 (6)** | **< 0.001** |
|  | Erosions (radiography) | 3/27 (11)^‡^ | 0/2 (0)* | 0/4 (0)* | NA | 0/6 (0)^ɫ^ | > 0.9 |
| Muscle involvement, *n/N (%)* | | **51/80 (64)** | 7/21 (33) | 6/21 (29) | 3/10 (30) | **16/52 (31)** | **< 0.001** |
|  | Muscle weakness (MRC ≤ 3) | 18/79 (23)^‡^ | 1/20 (5)* | 2/21 (10) | 3/10 (30) | 6/51 (12)^ɫ^ | 0.11 |
|  | Dysphagia | 5/80 (6) | 0/21 (0) | 0/21 (0) | 0/10 (0) | 0/52 (0) | 0.2 |
|  | Severe muscle involvement | **30/80 (38)** | 1/21 (5) | 2/21 (10) | 3/10 (30) | **6/52 (12)** | **0.001** |
| Fever, *n/N (%)* | | 22/79 (28)^‡^ | 10/21 (48) | 8/21 (38) | 4/10 (40) | 22/52 (42) | 0.09 |
| Pericarditis, *n/N (%)* | | 7/79 (9)^‡^ | 3/21 (14) | 2/21 (10) | 0/10 (0) | 5/52 (10) | > 0.9 |
| Myocarditis, *n/N (%)* | | 1/79 (1)^‡^ | 1/21 (5) | 0/21 (0) | 0/10 (0) | 1/52 (2) | > 0.9 |
| Gastroesophageal reflux, *n/N (%)* | | 9/78 (12)^‡^ | 2/21 (10) | 1/21 (5) | 0/10 (0) | 3/52 (6) | 0.4 |
| **Antibodies at ASyS diagnosis** | |  |  |  |  |  |  |
| Anti-Ro52 (TRIM21), *n/N (%)* | | 20/41(49)^‡^ | 7/12 (58)* | 9/14 (64)* | 4/8 (50)^ˠ^ | 20/34 (59)^ɫ^ | 0.5 |
| **Laboratory indicators at ILD diagnosis** | |  |  |  |  |  |  |
| C-reactive protein, mg/L^†^ | | **20 (6─46)** | 40 (19─77) | 48 (14─113) | 19 (10─30) | **31 (16─69)** | **0.032** |
| Ferritin, µg/L^†^ | | 355 (75─564) | 253 (230─521) | 215 (137─1206) | 545 (86─734) | 273 (138─545) | 0.8 |
| Creatine phosphokinase, IU/L^†^ | | **911 (190─2211)** | 496 (111─988) | 192 (58─900) | 171 (56─1200) | **264 (64─1200)** | **0.014** |
| **ILD outcomes at ILD diagnosis** | |  |  |  |  |  |  |
| Dyspnea, mMRC scale, *n/N (%)* | |  |  |  |  |  | 0.075 |
| 0 | | 13/63 (21)^‡^ | 2/18 (11)* | 4/16 (25)* | 0/7 (0)^ˠ^ | 6/41 (15)^ɫ^ |  |
| 1 | | 10/63 (16)^‡^ | 2/18 (11)* | 2/16 (13)* | 1/7 (14)^ˠ^ | 5/41 (12)^ɫ^ |  |
| 2 | | 16/63 (25)^‡^ | 2/18 (11)* | 3/16 (19)* | 2/7 (29)^ˠ^ | 7/41 (17)^ɫ^ |  |
| 3 | | 14/63 (22)^‡^ | 2/18 (11)* | 3/16 (19)* | 1/7 (14)^ˠ^ | 6/41 (15)^ɫ^ |  |
| 4 | | 10/63 (16)^‡^ | 10/18 (56)* | 4/16 (25)* | 3/7 (43)^ˠ^ | 17/41 (41)^ɫ^ |  |
| Cough, *n/N (%)* | | 41/78 (53)^‡^ | 13/20 (65)* | 14/19 (74)* | 7/10 (70) | 34/49 (69)^ɫ^ | 0.06 |
| ILD pattern, *n/N (%)* | |  |  |  |  |  |  |
|  | UIP | 3/43 (7)^‡^ | 5/15 (33)* | 0/9 (0)* | 0/4 (0)^ˠ^ | 5/28 (18)^ɫ^ | 0.2 |
|  | NSIP | 36/43 (84)^‡^ | 10/15 (67)* | 8/9 (89)* | 3/4 (75)^ˠ^ | 21/28 (75)^ɫ^ | 0.4 |
|  | OP | 6/43 (14)^‡^ | 3/15 (20)* | 1/9 (11)* | 1/4 (25)^ˠ^ | 5/28 (18)^ɫ^ | 0.7 |
| Pleural effusion, *n/N (%)* | | 10/72 (14)^‡^ | 6/20 (30)* | 5/21 (24) | 1/10 (10) | 12/51 (24)^ɫ^ | 0.2 |
| Pulmonary function test results | |  |  |  |  |  |  |
|  | PaO_2_, mmHg^†^ | 72 (64─90) | 75 (69─96) | 68 (61─83) | 63 (57─71) | 70 (63─90) | > 0.9 |
|  | FVC, % predicted^†^ | 74 (62─88) | 70 (64─88) | 67 (60─74) | 50 (45─72) | 67 (56─80) | 0.06 |
|  | TLC, % predicted^†^ | 77 (60─90) | 73 (61─90) | 71 (63─87) | 58 (49─60) | 66 (58─86) | 0.15 |
|  | DL_CO_, % predicted^†^ | 50 (42−68) | 54 (44─62) | 46 (29─56) | 47 (45─53) | 50 (41─58) | 0.4 |
|  | DL_CO_/VA, % predicted^†^ | 75 (64─85) | 80 (61─89) | 65 (52─72) | 92 (76─96) | 72 (61─86) | 0.6 |
| **ILD outcomes during follow-up** | |  |  |  |  |  |  |
| Number of ILD relapses^†^ | | 1 (0−2) | 1 (0−2) | 1 (0─3) | 1 (0─1) | 1 (0─2) | 0.4 |
| Estimated sPAP on TTE, mmHg^†^ | | 35 (28─42) | 29 (24─33) | 33 (25─44) | 37 (33─44) | 33 (25─44) | 0.8 |
| Peak TRV on TTE, m/s^†^ | | 2.6 (2.2─2.9) | 2.6 (2.4─2.8) | 2.4 (2.3─3) | 2.5 (2.3─3) | 2.5 (2.4─2.9) | 0.6 |
| mPAP on RHC, mmHg^†^ | | 26 (24─29) | 34 (34─34) | 35 (28─49) | 34 (34─34) | 34 (30─39) | 0.07 |
| Confirmed pulmonary hypertension, *n/N (%)* | | 5/80 (6) | 1/21 (5) | 4/21 (19) | 1/10 (10) | 6/52 (12) | 0.3 |
| Chronic respiratory failure, *n/N (%)* | | 15/79 (19)^‡^ | 3/21 (14) | 7/21 (33) | 4/10 (40) | 14/52 (27) | 0.3 |
| Long-term oxygen therapy, *n/N (%)* | | 14/78 (18)^‡^ | 3/21 (14) | 7/21 (33) | 3/10 (30) | 13/52 (25) | 0.3 |
| Lung transplantation, *n/N (%)* | | 0/80 (0) | 0/21 (0) | 1/21 (5) | 0/10 (0) | 1/52 (2) | 0.4 |
| Death, *n/N (%)* | | 14/80 (18) | 9/21 (43) | 3/21 (14) | 1/10 (10) | 13/52 (25) | 0.3 |
| **Treatments used during follow-up** | |  |  |  |  |  |  |
| Number of treatments used during follow-up^†^ | | 3 (2−4) | 2 (2─4) | 3 (2─4) | 3 (2─3) | 3 (2─4) | 0.2 |
| Corticosteroids, *n/N (%)* | | 75/80 (94) | 19/21 (90) | 21/21 (100) | 10/10 (100) | 50/52 (96) | 0.7 |
| Hydroxychloroquine, *n/N (%)* | | 10/80 (13) | 1/21 (5) | 2/21 (10) | 0/10 (0) | 3/52 (6) | 0.2 |
| Methotrexate, *n/N (%)* | | **40/80 (50)** | 4/21 (19) | 8/21 (38) | 2/10 (20) | **14/52 (27)** | **0.008** |
| Azathioprine, *n/N (%)* | | 22/80 (28) | 3/21 (14) | 6/21 (29) | 4/10 (40) | 13/52 (25) | 0.8 |
| Cyclophosphamide, *n/N (%)* | | 21/80 (26) | 6/21 (29) | 5/21 (24) | 4/10 (40) | 15/52 (29) | 0.7 |
| Mycophenolate mofetil, *n/N (%)* | | 30/80 (38) | 12/21 (57) | 8/21 (38) | 4/10 (40) | 24/52 (46) | 0.3 |
| Rituximab, *n/N (%)* | | 31/80 (39) | 5/21 (24) | 9/21 (43) | 5/10 (50) | 19/52 (37) | 0.8 |
| CNI, *n/N (%)* | | 9/80 (11) | 1/21 (5) | 2/21 (10) | 0/10 (0) | 3/52 (6) | 0.4 |
| IVIg, *n/N (%)* | | 18/80 (23) | 3/21 (14) | 2/21 (10) | 1/10 (10) | 6/52 (12) | 0.11 |
| Antifibrotic drugs, *n/N (%)* | | 3/80 (4) | 0/21 (0) | 2/21 (10) | 0/10 (0) | 2/52 (4) | > 0.9 |

Bold font indicates statistically significant results (P < 0.05). ^†^ Data are expressed as medians with interquartile ranges (IQRs). ^‡^ N ≠ 80 due to missing data. * N ≠ 21 due to missing data. ^ˠ^ N ≠ 10 because of missing data. ^ɫ^ N ≠ 52 because of missing data. **^ɪ^** Anti-Jo-1 patients were compared with non-anti-Jo-1 patients using Pearson's chi-square test, Wilcoxon rank sum test, Fisher's exact test or Wilcoxon rank sum exact test. ASyS: antisynthetase syndrome; BMI: body mass index; CNI: calcineurin inhibitor; DL_CO_: diffusion capacity of the lung for carbon monoxide; DL_CO_/VA: diffusion capacity of the lung for carbon monoxide per lung volume; FVC: forced vital capacity; ILD: interstitial lung disease; IVIg: intravenous immunoglobulin; mMRC: modified Medical Research Council dyspnea scale; mPAP: mean pulmonary arterial pressure; MRC: Medical Research Council muscle testing scale; NSIP: nonspecific interstitial pneumonia; NA: not available; OP: organized pneumonia; PaO2: arterial partial pressure of oxygen; RHC: right heart catheterization; sPAP: systolic pulmonary arterial pressure; TLC: total lung capacity; TRV: tricuspid regurgitation velocity; TTE: transthoracic echocardiography; UIP: usual interstitial pneumonia

**Supplementary Table S4.** Factors associated with all-cause mortality according to Cox regression analysis (after selection of clinically pertinent variables)

| **Variable** | **Univariate Cox regression** | | **Multivariate Cox regression** | |
| --- | --- | --- | --- | --- |
|  | **HR (95% CI)** | **P value** | **HR (95% CI)** | **P value** |
| **Demographics** |  |  |  |  |
| Male sex | **2.43 (1.09─5.41)** | **0.03** | 0.8 (0.11─5.6) | 0.82 |
| Age | **1.08 (1.05─1.12)** | **<0.001** | 1.1 (0.99─1.17) | 0.08 |
| BMI | **0.89 (0.81─0.97)** | **0.01** | **-** | **-** |
| Cancer-associated myositis | 2.21 (0.88─5.56) | 0.09 | 1.6 (0.52─4.8) | 0.41 |
| Overlapping connective tissue disease | 0.59 (0.18─1.98) | 0.39 | - | - |
| **Clinical manifestations at ASyS diagnosis** |  |  |  |  |
| Mechanic’s hands | **0.21 (0.05─0.089)** | **0.035** | **-** | **-** |
| Microvascular involvement | 0.58 (0.23─1.47) | 0.25 | **-** | **-** |
| Raynaud’s phenomenon | 0.69(0.26-1.85) | 0.46 | **-** | **-** |
| Joint involvement | **0.43 (0.19─0.98)** | **0.044** | **-** | **-** |
| Muscle involvement | **0.42 (0.18─0.99)** | **0.046** | 0.57 (0.17-1.97) | 0.38 |
| Severe muscle involvement | 0.35 (0.10─1.16) | 0.086 | **-** | **-** |
| Fever | **2.96 (1.32─6.66)** | **0.009** | **-** | **-** |
| Cardiac involvement (pericarditis and/or myocarditis) | **3.53 (1.40─8.91)** | **0.008** | **32.5 (4.11─257)** | **<0.001** |
| Gastroesophageal reflux | 1.48 (0.44─4.95) | 0.53 | **-** | **-** |
| **Antibodies at ASyS diagnosis** |  |  |  |  |
| Anti-Jo-1 antibodies | 0.71 (0.32─1.59) | 0.40 | **-** | **-** |
| Anti-PL-7 antibodies | **3.38 (1.44─7.91)** | **0.005** | 3.5 (0.78─15.8) | 0.10 |
| Anti-PL-12 antibodies | 0.43 (0.10─1.84) | 0.26 | - | - |
| Anti-Ro52 (TRIM21) antibodies | 0.60 (0.19─1.88) | 0.38 | - | - |
| **Laboratory indicators at ILD diagnosis** |  |  |  |  |
| C-reactive protein | 1 (1─1.01) | 0.44 | - | - |
| Ferritin | 0.78 (0.14─4.24) | 0.77 | - | - |
| **ILD outcomes at ILD diagnosis** |  |  |  |  |
| RP ILD  UIP pattern | 2.09 (0.94─4.68)  **3.95 (1.19─13.1)** | 0.07  **0.025** | 1.5 (0.30─7.2)  0.7 (0.08─5.45) | 0.64  0.71 |
| NSIP pattern | 0.35 (0.11─1.11) | 0.08 | - | - |
| FVC | 1 (0.98─1.02) | 0.71 | - | - |
| DL_CO_ | 0.99 (0.95─1.02) | 0.44 | - | - |
| Pleural effusion | **4.61 (2.01─10.6)** | **<0.001** | 3.3 (0.52─21.1) | 0.21 |
| **ILD outcomes during follow-up** |  |  |  |  |
| ILD relapse | 0.93 (0.71─1.22) | 0.61 | - | - |
| Chronic respiratory failure | 2.28 (0.99─5.27) | 0.054 | - | - |
| Long-term oxygen therapy | 2.08 (0.88─4.92) | 0.09 | - | - |
| **Treatments used during follow-up** |  |  |  |  |
| Number of treatments used | **0.66 (0.48-0.91)** | **0.011** | 0.5 (0.26-1.1) | 0.09 |
| Corticosteroids | **0.29 (0.09─0.99)** | **0.047** | - | - |
| Hydroxychloroquine | 0.33 (0.04─2.42) | 0.27 | - | - |
| Methotrexate | **0.31 (0.12─0.84)** | **0.02** | - | - |
| Azathioprine | 0.46 (0.16─1.36) | 0.16 | - | - |
| Cyclophosphamide | 1.11 (0.46─2.69) | 0.81 | - | - |
| Mycophenolate mofetil | **0.41 (0.16─1.05)** | **0.06** | - | - |
| CNI | 0.77 (0.18─3.27) | 0.72 | - | - |
| Rituximab | 0.46 (0.18─1.16) | 0.1 | - | - |
| IVIg | 0.99 (0.37─2.65) | 0.98 | - | - |

Bold font indicates statistically significant results (P < 0.05). ASyS: antisynthetase syndrome; BMI: body mass index; CI: confidence interval; CNI: calcineurin inhibitor; DL_CO_: diffusion capacity of the lung for carbon monoxide; FVC: forced vital capacity; HR: hazard ratio; ILD: interstitial lung disease; IVIg: intravenous immunoglobulins; NSIP: nonspecific interstitial pneumonia; RP ILD: rapidly progressive interstitial lung disease; UIP: usual interstitial pneumonia.

**Supplementary Table S5.** Comparison of clusters 1 and 2 derived from hierarchical clustering analysis in ASyS-ILD patients.

| **Variable** | | | **Cluster 1**  **(*N* = 62)** | **Cluster 2**  **(*N* = 40)** | **P-value** |
| --- | --- | --- | --- | --- | --- |
| **Demographics** | | |  |  |  |
| Female sex, *n/N (%)* | | | 44/62 (71) | 18/40 (45) | **0.017** |
| Age, *n/N (%)* | | |  |  | **<0.001** |
|  | ≤ 40 years | | 9/62 (15) | 0/40 (0) |  |
|  | 40-60 years | | 44/62 (71) | 8/40 (20) |  |
|  | > 60 years | | 9/62 (15) | 32/40 (80) |  |
| Comorbidities | | |  |  |  |
| Cancer-associated myositis, *n/N (%)* | | | 4/62 (7) | 10/40 (25) | **0.006** |
| Overlapping CTD, *n/N (%)* | | | 14/62 (23) | 2/40 (5) | **0.021** |
| **Clinical manifestations at ASyS diagnosis** | | |  |  |  |
| Skin involvement, *n/N (%)* | | |  |  |  |
|  | | Mechanic’s hands | 28/62 (45) | 5/40 (13) | **<0.001** |
|  | | Typical dermatomyositis signs | 11/62 (18) | 4/40 (10) | 0.3 |
| Microvascular involvement, *n/N (%)* | | | 29/62 (47) | 9/40 (23) | **0.02** |
| Joint involvement, *n/N (%)* | | | 49/62 (79) | 17/40 (43) |  |
| Muscle involvement, *n/N (%)* | | | 40/62 (65) | 18/40 (45) | **0.033** |
|  | | Severe muscle involvement | 24/62 (39) | 7/40 (18) | **0.01** |
| Fever, *n/N (%)* | | | 12/61 (20)^‡^ | 20/40 (50) | **0.004** |
| Pericarditis and/or myocarditis, *n/N (%)* | | | 3/61 (5)^‡^ | 7/40 (18) | **0.043** |
| **Antibodies at ASyS diagnosis** | | |  |  |  |
| Anti-Jo-1, *n/N (%)* | | | 57/62 (92) | 23/40 (58) | **<0.001** |
| Anti-PL-7, *n/N (%)* | | | 5/62 (8) | 16/40 (40) | **<0.001** |
| Anti-PL-12, *n/N (%)* | | | 0/62 (0) | 1/40 (3) | 0.4 |
| Anti-EJ, *n/N (%)* | | | 0/62 (0) | 0/40 (0) |  |
| Anti-Ro52 (TRIM21), *n/N (%)* | | | 17/34 (50)^‡^ | 10/20 (50)* | 0.8 |
| **Laboratory indicators at ILD diagnosis** | | |  |  |  |
| Creatine phosphokinase, *n/N (%)* | | |  |  | **0.003** |
|  | ≤ 170 IU/L | | 12/62 (19) | 17/40 (43) |  |
|  | 170-800 IU/L | | 16/62 (26) | 7/40 (18) |  |
|  | 800-2300 IU/L | | 14/62 (23) | 13/40 (33) |  |
|  | 2300-7000 IU/L | | 17/62 (27) | 3/40 (8) |  |
|  | > 7000 IU/L | | 3/62 (5) | 0/40 (0) |  |
| **ILD outcomes at ILD diagnosis** | | |  |  |  |
| Pleural effusion, *n/N (%)* | | | 1/55 (2)^‡^ | 16/38 (42)* | **<0.001** |
| FVC < 70% predicted, *n/N (%)* | | | 23/62 (37) | 20/40 (50) | 0.14 |
| FVC, % predicted^†^ | | | 79 (65─88) | 65 (54─84) | 0.053 |
| DL_CO_, % predicted† | | | 58 (44─71) | 45 (32─52) | **0.006** |
| RP-ILD at ILD diagnosis, *n/N (%)* | | | 11/62 (18) | 27/40 (68) | **<0.001** |
| **ILD outcomes during follow-up** | | |  |  |  |
| Number of ILD relapse^†^ | | | 0 (0─1) | 1 (0─2) | **0.011** |
| Suspected or confirmed PH, *n/N (%)* | | | 8/62 (13) | 11/40 (28) | 0.2 |
| Chronic respiratory failure, *n/N (%)* | | | 3/62 (5) | 15/39 (38)* | **<0.001** |
| Death and/or lung transplantation, *n/N (%)* | | | 2/62 (3) | 22/40 (55) | **<0.001** |
| **Number of treatments used during follow-up^†^** | | | 3 (2─4) | 3 (2─4) | 0.3 |

^†^ Data are expressed as the median and interquartile range (IQR). ^‡^ N ≠ 62 due to missing data. * N ≠ 40 due to missing data.

ASyS: antisynthetase syndrome; CTD: connective tissue disease; DL_CO_: diffusion capacity of the lung for carbon monoxide; FVC: forced vital capacity; ILD: interstitial lung disease; IQR: interquartile range; PH: pulmonary hypertension, RP ILD: rapidly progressive interstitial lung disease.

**Supplementary Table S6.** Comparison of clusters 1 and 3 derived from hierarchical clustering analysis in ASyS-ILD patients.

| **Variable** | | | **Cluster 1**  **(*N* = 62)** | **Cluster 3**  **(*N* = 20)** | **P-value** |
| --- | --- | --- | --- | --- | --- |
| **Demographics** | | |  |  |  |
| Female sex, *n/N (%)* | | | 44/62 (71) | 20/20 (100) | **0.004** |
| Age, *n/N (%)* | | |  |  | **0.13** |
|  | ≤ 40 years | | 9/62 (15) | 6/20 (30) |  |
|  | 40-60 years | | 44/62 (71) | 9/20 (45) |  |
|  | > 60 years | | 9/62 (15) | 5/20 (25) |  |
| Comorbidities | | |  |  |  |
| Cancer-associated myositis, *n/N (%)* | | | 4/62 (7) | 2/20 (10) | 0.6 |
| Overlapping CTD, *n/N (%)* | | | 14/62 (23) | 6/20 (30) | 0.6 |
| **Clinical manifestations at ASyS diagnosis** | | |  |  |  |
| Skin involvement, *n/N (%)* | | |  |  |  |
|  | | Mechanic’s hands | 28/62 (45) | 6/20 (30) | 0.3 |
|  | | Typical dermatomyositis signs | 11/62 (18) | 6/20 (30) | 0.3 |
| Microvascular involvement, *n/N (%)* | | | 29/62 (47) | 7/20 (35) | 0.4 |
| Joint involvement, *n/N (%)* | | | 49/62 (79) | 10/20 (50) | **0.017** |
| Muscle involvement, *n/N (%)* | | | 40/62 (65) | 6/20 (30) | **0.006** |
|  | | Severe muscle involvement | 24/62 (39) | 2/20 (10) | **0.014** |
| Fever, *n/N (%)* | | | 12/61 (20)^‡^ | 8/20 (40) | 0.09 |
| Pericarditis and/or myocarditis, *n/N (%)* | | | 3/61 (5)^‡^ | 2/20 (10) | 0.6 |
| **Antibodies at ASyS diagnosis** | | |  |  |  |
| Anti-Jo-1, *n/N (%)* | | | 57/62 (92) | 0/20 (0) | **<0.001** |
| Anti-PL-7, *n/N (%)* | | | 5/62 (8) | 0/20 (0) | 0.3 |
| Anti-PL-12, *n/N (%)* | | | 0/62 (0) | 20/20 (100) | **<0.001** |
| Anti-EJ, *n/N (%)* | | | 0/62 (0) | 0/20 (0) |  |
| Anti-Ro52 (TRIM21), *n/N (%)* | | | 17/34 (50)^‡^ | 9/13 (69)^ɫ^ | 0.2 |
| **Laboratory indicators at ILD diagnosis** | | |  |  |  |
| Creatine phosphokinase, *n/N (%)* | | |  |  | 0.2 |
|  | ≤ 170 IU/L | | 12/62 (19) | 9/20 (45) |  |
|  | 170-800 IU/L | | 16/62 (26) | 5/20 (25) |  |
|  | 800-2300 IU/L | | 14/62 (23) | 4/20 (20) |  |
|  | 2300-7000 IU/L | | 17/62 (27) | 2/20 (10) |  |
|  | > 7000 IU/L | | 3/62 (5) | 0/20 (0) |  |
| **ILD outcomes at ILD diagnosis** | | |  |  |  |
| Pleural effusion, *n/N (%)* | | | 1/55 (2)^‡^ | 4/20 (20) | **0.016** |
| FVC < 70% predicted, *n/N (%)* | | | 23/62 (37) | 10/20 (50) | 0.3 |
| FVC, % predicted^†^ | | | 79 (65─88) | 69 (61─77) | 0.057 |
| DL_CO_, % predicted† | | | 58 (44─71) | 48 (41─56) | 0.13 |
| RP-ILD at ILD diagnosis, *n/N (%)* | | | 11/62 (18) | 8/20 (40) | 0.063 |
| **ILD outcomes during follow-up** | | |  |  |  |
| Number of ILD relapse^†^ | | | 0 (0─1) | 2 (0─3) | 0.095 |
| Suspected or confirmed PH, *n/N (%)* | | | 8/62 (13) | 5/20 (25) | 0.3 |
| Chronic respiratory failure, *n/N (%)* | | | 3/62 (5) | 7/20 (35) | **0.001** |
| Death and/or lung transplantation, *n/N (%)* | | | 2/62 (3) | 3/20 (15) | 0.088 |
| **Number of treatments used during follow-up^†^** | | | 3 (2─4) | 3 (2─4) | >0.9 |

^†^ Data are expressed as the median and interquartile range (IQR). ^‡^ N ≠ 62 due to missing data. ^ɫ^ N ≠ 20 because of missing data.

ASyS: antisynthetase syndrome; CTD: connective tissue disease; FVC: forced vital capacity; ILD: interstitial lung disease; IQR: interquartile range; PH: pulmonary hypertension, RP ILD: rapidly progressive interstitial lung disease.

**Supplementary Table S7.** Comparison of clusters 1 and 4 derived from hierarchical clustering analysis in ASyS-ILD patients.

| **Variable** | | | **Cluster 1**  **(*N* = 62)** | **Cluster 4**  **(*N* = 10)** | **P-value** |
| --- | --- | --- | --- | --- | --- |
| **Demographics** | | |  |  |  |
| Female sex, *n/N (%)* | | | 44/62 (71) | 9/10 (90) | 0.3 |
| Age, *n/N (%)* | | |  |  | 0.12 |
|  | ≤ 40 years | | 9/62 (15) | 0/10 (0) |  |
|  | 40-60 years | | 44/62 (71) | 6/10 (60) |  |
|  | > 60 years | | 9/62 (15) | 4/10 (40) |  |
| Comorbidities | | |  |  |  |
| Cancer-associated myositis, *n/N (%)* | | | 4/62 (7) | 2/10 (20) | 0.2 |
| Overlapping CTD, *n/N (%)* | | | 14/62 (23) | 2/10 (20) | >0.9 |
| **Clinical manifestations at ASyS diagnosis** | | |  |  |  |
| Skin involvement, *n/N (%)* | | |  |  |  |
|  | | Mechanic’s hands | 28/62 (45) | 2/10 (20) | 0.2 |
|  | | Typical dermatomyositis signs | 11/62 (18) | 0/10 (0) | 0.3 |
| Microvascular involvement, *n/N (%)* | | | 29/62 (47) | 2/10 (20) | 0.2 |
| Joint involvement, *n/N (%)* | | | 49/62 (79) | 2/10 (20) | **<0.001** |
| Muscle involvement, *n/N (%)* | | | 40/62 (65) | 3/10 (30) | 0.078 |
|  | | Severe muscle involvement | 24/62 (39) | 3/10 (30) | 0.7 |
| Fever, *n/N (%)* | | | 12/61 (20)^‡^ | 4/10 (40) | 0.2 |
| Pericarditis and/or myocarditis, *n/N (%)* | | | 3/61 (5)^‡^ | 0/10 (0) | >0.9 |
| **Antibodies at ASyS diagnosis** | | |  |  |  |
| Anti-Jo-1, *n/N (%)* | | | 57/62 (92) | 0/10 (0) | **<0.001** |
| Anti-PL-7, *n/N (%)* | | | 5/62 (8) | 0/10 (0) | >0.9 |
| Anti-PL-12, *n/N (%)* | | | 0/62 (0) | 0/10 (0) |  |
| Anti-EJ, *n/N (%)* | | | 0/62 (0) | 10/10 (100) | **<0.001** |
| Anti-Ro52 (TRIM21), *n/N (%)* | | | 17/34 (50)^‡^ | 4/8 (50)^ˠ^ | >0.9 |
| **Laboratory indicators at ILD diagnosis** | | |  |  |  |
| Creatine phosphokinase, *n/N (%)* | | |  |  | 0.3 |
|  | ≤ 170 IU/L | | 12/62 (19) | 5/10 (50) |  |
|  | 170-800 IU/L | | 16/62 (26) | 1/10 (10) |  |
|  | 800-2300 IU/L | | 14/62 (23) | 3/10 (30) |  |
|  | 2300-7000 IU/L | | 17/62 (27) | 1/10 (10) |  |
|  | > 7000 IU/L | | 3/62 (5) | 0/10 (0) |  |
| **ILD outcomes at ILD diagnosis** | | |  |  |  |
| Pleural effusion, *n/N (%)* | | | 1/55 (2)^‡^ | 1/10 (10) | 0.3 |
| FVC < 70% predicted, *n/N (%)* | | | 23/62 (37) | 7/10 (70) | 0.08 |
| FVC, % predicted^†^ | | | 79 (65─88) | 50 (45─72) | **0.005** |
| DL_CO_, % predicted† | | | 58 (44─71) | 47 (45─53) | 0.3 |
| RP-ILD at ILD diagnosis, *n/N (%)* | | | 11/62 (18) | 5/10 (50) | **0.035** |
| **ILD outcomes during follow-up** | | |  |  |  |
| Number of ILD relapse^†^ | | | 0 (0─1) | 1 (0─1) | 0.8 |
| Suspected or confirmed PH, *n/N (%)* | | | 8/62 (13) | 3/10 (30) | 0.4 |
| Chronic respiratory failure, *n/N (%)* | | | 3/62 (5) | 4/10 (40) | **0.005** |
| Death and/or lung transplantation, *n/N (%)* | | | 2/62 (3) | 1/10 (10) | 0.4 |
| **Number of treatments used during follow-up^†^** | | | 3 (2─4) | 3 (2─3) | 0.6 |

^†^ Data are expressed as the median and interquartile range (IQR). ^‡^ N ≠ 62 due to missing data. ^ˠ^ N ≠ 10 because of missing data.

ASyS: antisynthetase syndrome; CTD: connective tissue disease; FVC: forced vital capacity; ILD: interstitial lung disease; IQR: interquartile range; PH: pulmonary hypertension, RP ILD: rapidly progressive interstitial lung disease.

**Supplementary Table S8.** Comparison of clusters 2 and 3 derived from hierarchical clustering analysis in ASyS-ILD patients.

| **Variable** | | | **Cluster 2**  **(*N* = 40)** | **Cluster 3**  **(*N* = 20)** | **P-value** |
| --- | --- | --- | --- | --- | --- |
| **Demographics** | | |  |  |  |
| Female sex, *n/N (%)* | | | 18/40 (45) | 20/20 (100) | **<0.001** |
| Age, *n/N (%)* | | |  |  | **<0.001** |
|  | ≤ 40 years | | 0/40 (0) | 6/20 (30) |  |
|  | 40-60 years | | 8/40 (20) | 9/20 (45) |  |
|  | > 60 years | | 32/40 (80) | 5/20 (25) |  |
| Comorbidities | | |  |  |  |
| Cancer-associated myositis, *n/N (%)* | | | 10/40 (25) | 2/20 (10) | 0.2 |
| Overlapping CTD, *n/N (%)* | | | 2/40 (5) | 6/20 (30) | **0.014** |
| **Clinical manifestations at ASyS diagnosis** | | |  |  |  |
| Skin involvement, *n/N (%)* | | |  |  |  |
|  | | Mechanic’s hands | 5/40 (13) | 6/20 (30) | 0.2 |
|  | | Typical dermatomyositis signs | 4/40 (10) | 6/20 (30) | 0.074 |
| Microvascular involvement, *n/N (%)* | | | 9/40 (23) | 7/20 (35) | 0.3 |
| Joint involvement, *n/N (%)* | | | 17/40 (43) | 10/20 (50) | 0.6 |
| Muscle involvement, *n/N (%)* | | | 18/40 (45) | 6/20 (30) | 0.3 |
|  | | Severe muscle involvement | 7/40 (18) | 2/20 (10) | 0.7 |
| Fever, *n/N (%)* | | | 20/40 (50) | 8/20 (40) | 0.5 |
| Pericarditis and/or myocarditis, *n/N (%)* | | | 7/40 (18) | 2/20 (10) | 0.7 |
| **Antibodies at ASyS diagnosis** | | |  |  |  |
| Anti-Jo-1, *n/N (%)* | | | 23/40 (58) | 0/20 (0) | **<0.001** |
| Anti-PL-7, *n/N (%)* | | | 16/40 (40) | 0/20 (0) | **<0.001** |
| Anti-PL-12, *n/N (%)* | | | 1/40 (3) | 20/20 (100) | **<0.001** |
| Anti-EJ, *n/N (%)* | | | 0/40 (0) | 0/20 (0) |  |
| Anti-Ro52 (TRIM21), *n/N (%)* | | | 10/20 (50)* | 9/13 (69)^ɫ^ | 0.3 |
| **Laboratory indicators at ILD diagnosis** | | |  |  |  |
| Creatine phosphokinase, *n/N (%)* | | |  |  | 0.6 |
|  | ≤ 170 IU/L | | 17/40 (43) | 9/20 (45) |  |
|  | 170-800 IU/L | | 7/40 (18) | 5/20 (25) |  |
|  | 800-2300 IU/L | | 13/40 (33) | 4/20 (20) |  |
|  | 2300-7000 IU/L | | 3/40 (8) | 2/20 (10) |  |
|  | > 7000 IU/L | | 0/40 (0) | 0/20 (0) |  |
| **ILD outcomes at ILD diagnosis** | | |  |  |  |
| Pleural effusion, *n/N (%)* | | | 16/38 (42)* | 4/20 (20) | 0.079 |
| FVC < 70% predicted, *n/N (%)* | | | 20/40 (50) | 10/20 (50) | >0.9 |
| FVC, % predicted^†^ | | | 65 (54─84) | 69 (61─77) | >0.9 |
| DL_CO_, % predicted† | | | 45 (32─52) | 48 (41─56) | 0.7 |
| RP-ILD at ILD diagnosis, *n/N (%)* | | | 27/40 (68) | 8/20 (40) | **0.03** |
| **ILD outcomes during follow-up** | | |  |  |  |
| Number of ILD relapse^†^ | | | 1 (0─2) | 2 (0─3) | >0.9 |
| Suspected or confirmed PH, *n/N (%)* | | | 11/40 (28) | 5/20 (25) | >0.9 |
| Chronic respiratory failure, *n/N (%)* | | | 15/39 (38)* | 7/20 (35) | 0.7 |
| Death and/or lung transplantation, *n/N (%)* | | | 22/40 (55) | 3/20 (15) | **0.002** |
| **Number of treatments used during follow-up^†^** | | | 3 (2─4) | 3 (2─4) | 0.3 |

^†^ Data are expressed as the median and interquartile range (IQR). * N ≠ 40 due to missing data. ^ɫ^ N ≠ 20 because of missing data.

ASyS: antisynthetase syndrome; CTD: connective tissue disease; FVC: forced vital capacity; ILD: interstitial lung disease; IQR: interquartile range; PH: pulmonary hypertension, RP ILD: rapidly progressive interstitial lung disease.

**Supplementary Table S9.** Comparison of clusters 2 and 4 derived from hierarchical clustering analysis in ASyS-ILD patients.

| **Variable** | | | **Cluster 2**  **(*N* = 40)** | **Cluster 4**  **(*N* = 10)** | **P-value** |
| --- | --- | --- | --- | --- | --- |
| **Demographics** | | |  |  |  |
| Female sex, *n/N (%)* | | | 18/40 (45) | 9/10 (90) | **0.015** |
| Age, *n/N (%)* | | |  |  | **0.022** |
|  | ≤ 40 years | | 0/40 (0) | 0/10 (0) |  |
|  | 40-60 years | | 8/40 (20) | 6/10 (60) |  |
|  | > 60 years | | 32/40 (80) | 4/10 (40) |  |
| Comorbidities | | |  |  |  |
| Cancer-associated myositis, *n/N (%)* | | | 10/40 (25) | 2/10 (20) | >0.9 |
| Overlapping CTD, *n/N (%)* | | | 2/40 (5) | 2/10 (20) | 0.2 |
| **Clinical manifestations at ASyS diagnosis** | | |  |  |  |
| Skin involvement, *n/N (%)* | | |  |  |  |
|  | | Mechanic’s hands | 5/40 (13) | 2/10 (20) | 0.6 |
|  | | Typical dermatomyositis signs | 4/40 (10) | 0/10 (0) | 0.6 |
| Microvascular involvement, *n/N (%)* | | | 9/40 (23) | 2/10 (20) | >0.9 |
| Joint involvement, *n/N (%)* | | | 17/40 (43) | 2/10 (20) | 0.3 |
| Muscle involvement, *n/N (%)* | | | 18/40 (45) | 3/10 (30) | 0.5 |
|  | | Severe muscle involvement | 7/40 (18) | 3/10 (30) | 0.4 |
| Fever, *n/N (%)* | | | 20/40 (50) | 4/10 (40) | 0.7 |
| Pericarditis and/or myocarditis, *n/N (%)* | | | 7/40 (18) | 0/10 (0) | 0.3 |
| **Antibodies at ASyS diagnosis** | | |  |  |  |
| Anti-Jo-1, *n/N (%)* | | | 23/40 (58) | 0/10 (0) | **0.001** |
| Anti-PL-7, *n/N (%)* | | | 16/40 (40) | 0/10 (0) | **0.02** |
| Anti-PL-12, *n/N (%)* | | | 1/40 (3) | 0/10 (0) | >0.9 |
| Anti-EJ, *n/N (%)* | | | 0/40 (0) | 10/10 (100) | **<0.001** |
| Anti-Ro52 (TRIM21), *n/N (%)* | | | 10/20 (50)* | 4/8 (50)^ˠ^ | >0.9 |
| **Laboratory indicators at ILD diagnosis** | | |  |  |  |
| Creatine phosphokinase, *n/N (%)* | | |  |  | 0.9 |
|  | ≤ 170 IU/L | | 17/40 (43) | 5/10 (50) |  |
|  | 170-800 IU/L | | 7/40 (18) | 1/10 (10) |  |
|  | 800-2300 IU/L | | 13/40 (33) | 3/10 (30) |  |
|  | 2300-7000 IU/L | | 3/40 (8) | 1/10 (10) |  |
|  | > 7000 IU/L | | 0/40 (0) | 0/10 (0) |  |
| **ILD outcomes at ILD diagnosis** | | |  |  |  |
| Pleural effusion, *n/N (%)* | | | 16/38 (42)* | 1/10 (10) | **0.07** |
| FVC < 70% predicted, *n/N (%)* | | | 20/40 (50) | 7/10 (70) | 0.5 |
| FVC, % predicted^†^ | | | 65 (54─84) | 50 (45─72) | 0.094 |
| DL_CO_, % predicted† | | | 45 (32─52) | 47 (45─53) | 0.5 |
| RP-ILD at ILD diagnosis, *n/N (%)* | | | 27/40 (68) | 5/10 (50) | 0.3 |
| **ILD outcomes during follow-up** | | |  |  |  |
| Number of ILD relapse^†^ | | | 1 (0─2) | 1 (0─1) | 0.2 |
| Suspected or confirmed PH, *n/N (%)* | | | 11/40 (28) | 3/10 (30) | >0.9 |
| Chronic respiratory failure, *n/N (%)* | | | 15/39 (38)* | 4/10 (40) | >0.9 |
| Death and/or lung transplantation, *n/N (%)* | | | 22/40 (55) | 1/10 (10) | **0.012** |
| **Number of treatments used during follow-up^†^** | | | 3 (2─4) | 3 (2─3) | >0.9 |

^†^ Data are expressed as the median and interquartile range (IQR). * N ≠ 40 due to missing data. ^ˠ^ N ≠ 10 because of missing data.

ASyS: antisynthetase syndrome; CTD: connective tissue disease; FVC: forced vital capacity; ILD: interstitial lung disease; IQR: interquartile range; PH: pulmonary hypertension, RP ILD: rapidly progressive interstitial lung disease.

**Supplementary Table S10.** Comparison of clusters 3 and 4 derived from hierarchical clustering analysis in ASyS-ILD patients.

| **Variable** | | | **Cluster 3**  **(*N* = 20)** | **Cluster 4**  **(*N* = 10)** | **P-value** |
| --- | --- | --- | --- | --- | --- |
| **Demographics** | | |  |  |  |
| Female sex, *n/N (%)* | | | 20/20 (100) | 9/10 (90) | 0.3 |
| Age, *n/N (%)* | | |  |  | 0.2 |
|  | ≤ 40 years | | 6/20 (30) | 0/10 (0) |  |
|  | 40-60 years | | 9/20 (45) | 6/10 (60) |  |
|  | > 60 years | | 5/20 (25) | 4/10 (40) |  |
| Comorbidities | | |  |  |  |
| Cancer-associated myositis, *n/N (%)* | | | 2/20 (10) | 2/10 (20) | 0.6 |
| Overlapping CTD, *n/N (%)* | | | 6/20 (30) | 2/10 (20) | 0.7 |
| **Clinical manifestations at ASyS diagnosis** | | |  |  |  |
| Skin involvement, *n/N (%)* | | |  |  |  |
|  | | Mechanic’s hands | 6/20 (30) | 2/10 (20) | 0.7 |
|  | | Typical dermatomyositis signs | 6/20 (30) | 0/10 (0) | 0.074 |
| Microvascular involvement, *n/N (%)* | | | 7/20 (35) | 2/10 (20) | 0.7 |
| Joint involvement, *n/N (%)* | | | 10/20 (50) | 2/10 (20) | 0.2 |
| Muscle involvement, *n/N (%)* | | | 6/20 (30) | 3/10 (30) | >0.9 |
|  | | Severe muscle involvement | 2/20 (10) | 3/10 (30) | 0.3 |
| Fever, *n/N (%)* | | | 8/20 (40) | 4/10 (40) | >0.9 |
| Pericarditis and/or myocarditis, *n/N (%)* | | | 2/20 (10) | 0/10 (0) | 0.5 |
| **Antibodies at ASyS diagnosis** | | |  |  |  |
| Anti-Jo-1, *n/N (%)* | | | 0/20 (0) | 0/10 (0) |  |
| Anti-PL-7, *n/N (%)* | | | 0/20 (0) | 0/10 (0) |  |
| Anti-PL-12, *n/N (%)* | | | 20/20 (100) | 0/10 (0) | **<0.001** |
| Anti-EJ, *n/N (%)* | | | 0/20 (0) | 10/10 (100) | **<0.001** |
| Anti-Ro52 (TRIM21), *n/N (%)* | | | 9/13 (69)^ɫ^ | 4/8 (50)^ˠ^ | 0.6 |
| **Laboratory indicators at ILD diagnosis** | | |  |  |  |
| Creatine phosphokinase, *n/N (%)* | | |  |  | 0.9 |
|  | ≤ 170 IU/L | | 9/20 (45) | 5/10 (50) |  |
|  | 170-800 IU/L | | 5/20 (25) | 1/10 (10) |  |
|  | 800-2300 IU/L | | 4/20 (20) | 3/10 (30) |  |
|  | 2300-7000 IU/L | | 2/20 (10) | 1/10 (10) |  |
|  | > 7000 IU/L | | 0/20 (0) | 0/10 (0) |  |
| **ILD outcomes at ILD diagnosis** | | |  |  |  |
| Pleural effusion, *n/N (%)* | | | 4/20 (20) | 1/10 (10) | 0.6 |
| FVC < 70% predicted, *n/N (%)* | | | 10/20 (50) | 7/10 (70) | 0.4 |
| FVC, % predicted^†^ | | | 69 (61─77) | 50 (45─72) | 0.12 |
| DL_CO_, % predicted† | | | 48 (41─56) | 47 (45─53) | >0.9 |
| RP-ILD at ILD diagnosis, *n/N (%)* | | | 8/20 (40) | 5/10 (50) | 0.7 |
| **ILD outcomes during follow-up** | | |  |  |  |
| Number of ILD relapse^†^ | | | 2 (0─3) | 1 (0─1) | 0.3 |
| Suspected or confirmed PH, *n/N (%)* | | | 5/20 (25) | 3/10 (30) | >0.9 |
| Chronic respiratory failure, *n/N (%)* | | | 7/20 (35) | 4/10 (40) | >0.9 |
| Death and/or lung transplantation, *n/N (%)* | | | 3/20 (15) | 1/10 (10) | >0.9 |
| **Number of treatments used during follow-up^†^** | | | 3 (2─4) | 3 (2─3) | 0.5 |

^†^ Data are expressed as the median and interquartile range (IQR). ^ɫ^ N ≠ 20 because of missing data. ^ˠ^ N ≠ 10 because of missing data.

ASyS: antisynthetase syndrome; CTD: connective tissue disease; FVC: forced vital capacity; ILD: interstitial lung disease; IQR: interquartile range; PH: pulmonary hypertension, RP ILD: rapidly progressive interstitial lung disease.
