# Supplementary figures and images for: Clinical profiles associated with rapidly progressive interstitial lung disease in antisynthetase syndrome: A multicentric cohort study (TYPASS study)
Source: J Intern Med. 2025 Dec 10;299(3):365–80. doi: 10.1111/joim.70058 (PMC12869006; doi:10.1111/joim.70058)

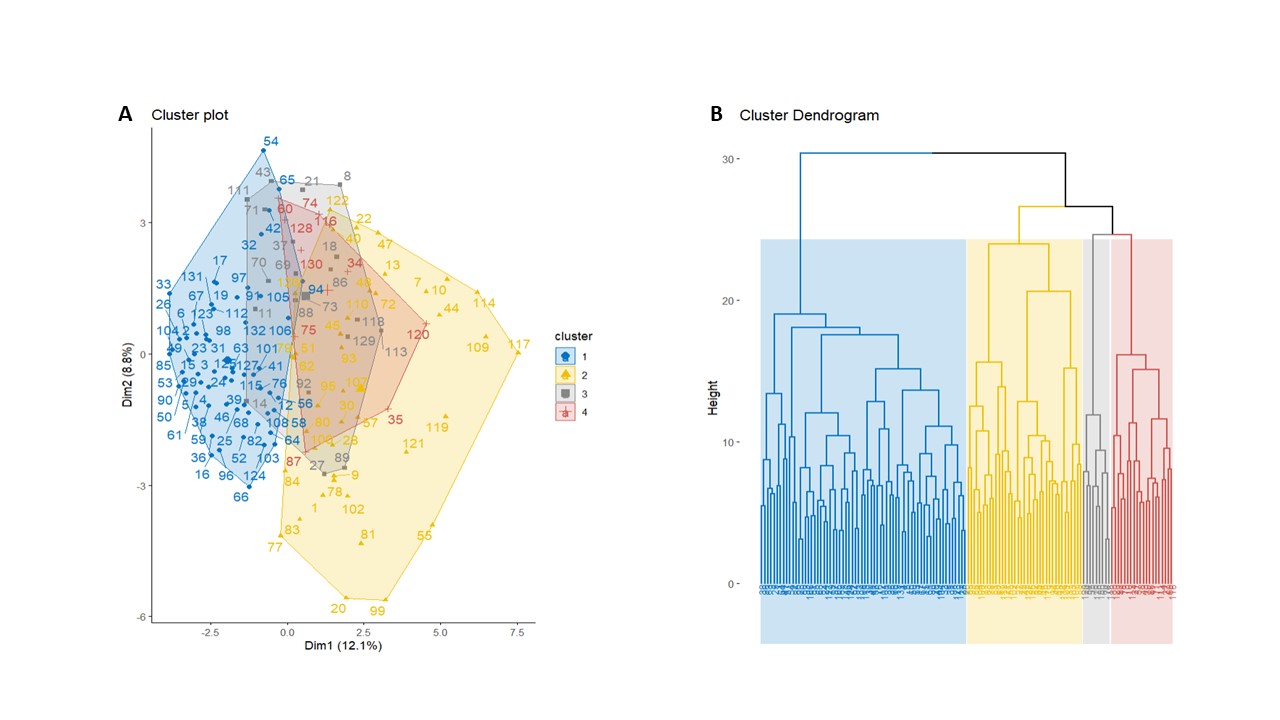

Supplement: Supplementary file 2 — Figure S1: Hierarchical clustering analysis of ASyS‐ILD patients according to included variables at baseline and during follow‐up (cumulative data). (A) Multiple correspondence analysis factor map. Factor map showing the row data (patients) used to generate the dendrogram. Dimensions 1 and 2 cumulatively explained 20.9% of the total variance. (B) Dendrogram. The y‐axis indicates the height of fusion into the clusters proposed, and the x‐axis indicates ASyS‐ILD patients (n = 132). A hierarchical tree indicates ASyS‐ILD patients according to the cluster to which they belong. Alt text: Visual representation of the four clusters derived from unsupervised analysis (panel A displays a factor map, and panel B presents a dendrogram). ASyS, antisynthetase syndrome; ILD, interstitial lung disease. [file JOIM-299-365-s001.jpg]

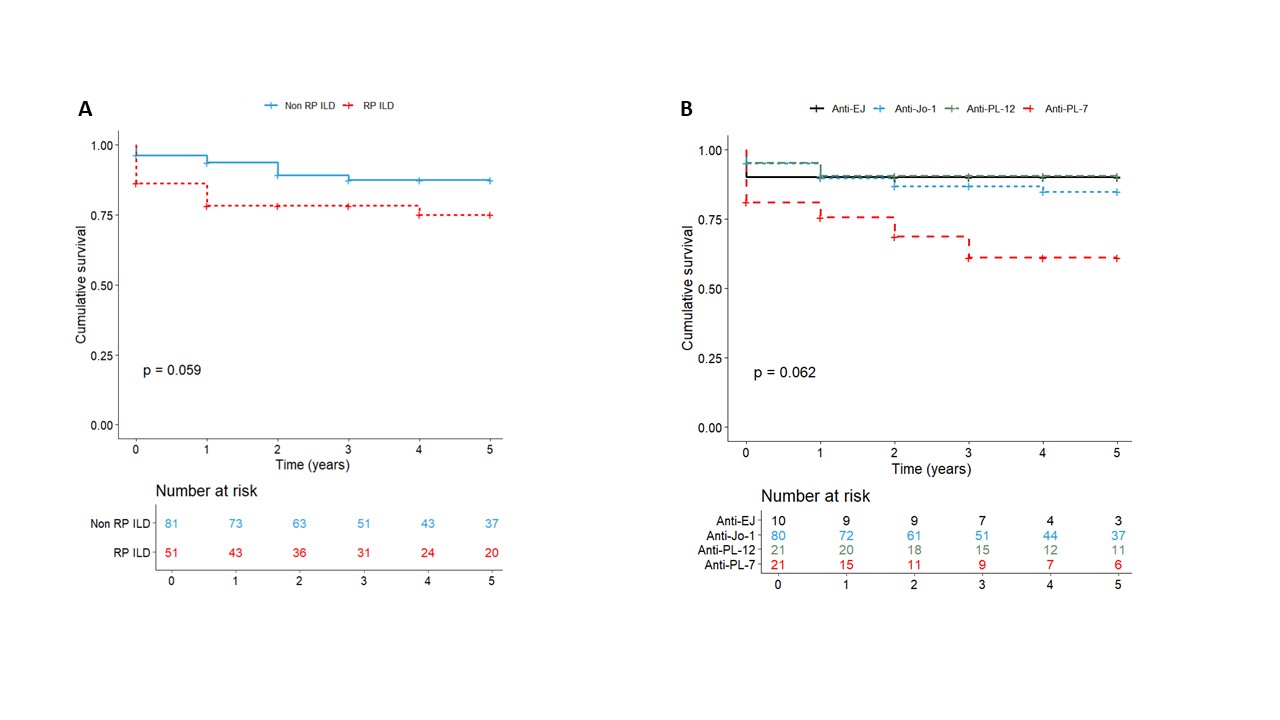

Supplement: Supplementary file 3 — Figure S2: Overall survival or transplant‐free survival from the time of ILD diagnosis according to rapidly progressive ILD at time of ILD diagnosis (A) and anti‐ARS antibodies (B). Alt text: Kaplan–Meier survival curves comparing patients with rapidly progressive interstitial lung disease (RP‐ILD) versus those without (panel A) and patients stratified by anti‐aminoacyl‐tRNA synthetase antibody status (panel B), indicating a trend toward worse survival in RP‐ILD patients and those positive for anti‐PL‐7 antibodies. [file JOIM-299-365-s003.jpg]
